# Combination LIGHT overexpression and checkpoint blockade disrupts the tumor immune environment impacting colorectal liver metastases

**DOI:** 10.1126/sciadv.adv9161

**Published:** 2025-10-08

**Authors:** Bridget P. Keenan, Guilin Qiao, Nicholas Kunda, Lyonell Kone, Sophia M. Guldberg, Letizia Todeschini, Prabhakaran Kumar, Tommaso Pollini, Sophia Hernandez, Jianzhong Qin, Lawrence Fong, Matthew H. Spitzer, Bellur S. Prabhakar, Ajay V. Maker

**Affiliations:** ^1^ Department of Medicine, Division of Hematology/Oncology, University of California, San Francisco, San Francisco, CA 94143, USA.; ^2^ Department of Surgery, Division of Surgical Oncology, University of California, San Francisco, San Francisco, CA 94143, USA.; ^3^ Department of Surgery, Division of Surgical Oncology, University of Illinois at Chicago, College of Medicine, Chicago, IL 60612, USA.; ^4^ Departments of Otolaryngology-Head and Neck Surgery and Microbiology and Immunology, University of California, San Francisco, San Francisco, CA 94143, USA.; ^5^ Department of Microbiology & Immunology, University of Illinois at Chicago, College of Medicine, Chicago, IL 60612, USA.; ^6^ Parker Institute for Cancer Immunotherapy, San Francisco, CA 94129, USA.; ^7^ Chan Zuckerberg Biohub, San Francisco, CA 94158, USA.

## Abstract

Colorectal cancer and liver metastases are a leading cause of cancer-related mortality. Overexpression of the immunostimulatory cytokine TNFSF14/LIGHT associates with improved survival and correlates with increased tumor-infiltrating lymphocytes in patients and a clinically relevant model of colorectal liver metastases. We demonstrate that LIGHT monotherapy activates T cells, but also induces T cell exhaustion and the recruitment of immunosuppressive elements. As colorectal liver metastases exhibit high levels of CTLA-4 expression, we combined LIGHT overexpression with anti-CTLA-4, leading to complete tumor control. The combination functions by homing tumor-infiltrating lymphocytes, inducing tumor antigen-specific T cells, and reversing T cell exhaustion. Whereas both LIGHT overexpression and anti-CTLA-4 increase tumor-promoting macrophages, the combination eliminates this population. The ability of LIGHT overexpression combined with CTLA-4 inhibition to reverse T cell exhaustion and myeloid cell suppression is supported by analysis of complementary patient cohorts and has strong clinical relevance, especially given that liver metastases contribute to immunotherapy resistance across various cancer types.

## Teaser

The combination of LIGHT with anti–CTLA-4 controls colorectal cancer liver metastases by reshaping the tumor microenvironment.

## INTRODUCTION

Colorectal cancer (CRC) is the third leading site of new cancer cases and is the second leading cause of cancer deaths annually in the United States (*1*). The liver is the most common site of CRC metastasis, and liver metastases will occur in up to 50% of all patients during the course of their disease. Unfortunately, long-term survival even after surgical resection of liver metastases remains elusive (*2*). Although we and others have demonstrated unprecedented responses with immune checkpoint blockade (ICB) in other cancers (*3*, *4*), ICB does not impart clinically meaningful responses in microsatellite-stable (MSS) colon cancer, which represents >95% of colon cancers. These tumors have a lower neoantigen load than microsatellite-instable (MSI-H) CRC and, unlike MSI-H CRC, rarely respond to checkpoint blockade alone or in combination (*5*, *6*). Similarly, adoptive transfer of CRC antigen-specific T cells has resulted in limited efficacy as well as dose-limiting toxicity (*7*, *8*). Furthermore, clinical trials have demonstrated that the response rates to ICB are lowest when liver metastases are present (*9*). Thus, there is a need to address this critical gap in knowledge to develop novel immunotherapy combinations that can overcome treatment resistance in MSS CRC metastatic cancers.

LIGHT (TNFSF14) is an immunostimulatory cytokine required for activation of CD8^+^ T cells that augments the antitumor immune response and overcomes the stromal antigenic barrier (*10*, *11*). Enhanced expression of LIGHT in the tumor microenvironment (TME) generates intratumoral tertiary lymphoid structures (TLSs) representative of an advanced immune response (*12*). We previously evaluated surgically resected colorectal liver metastases (CRLMs) from our patients and determined that increased LIGHT expression in the TME associated with increased T cell proliferation and activation and improved overall survival (OS) and recurrence-free survival (RFS) (*13*). In an additional cohort of patients with CRLMs, we determined that increased LIGHT^+^ tumor-infiltrating lymphocytes (TILs) were associated with improved OS, further supporting the premise that increasing LIGHT expression in CRLMs may improve antitumor immune responses and clinical outcomes (*13*, *14*). In an MSS immunocompetent CRLM preclinical model, we found that TIL activation alone was insufficient to entirely prevent metastatic tumor progression (*15*, *16*). By up-regulating LIGHT expression within the TME, we were able to partially overcome this inhibition threshold and drive antitumor immune responses, primarily driven by CD8^+^ T cells (*15*). Nevertheless, unidentified inhibitory signals persisted, preventing complete tumor regression with LIGHT monotherapy. We hypothesize that immune activation with LIGHT/TNFSF14 alone, like with other immunostimulants, may lead to T cell exhaustion and/or support of immunosuppressive elements in the TME. Because a large proportion of CRC TILs are CTLA-4^+^ at baseline, inhibited by CTLA-4:B7 engagement (*17*), and LIGHT traffics CTLA-4^+^ T cells into the tumor, we hypothesized that a combination of LIGHT and anti–CTLA-4 therapy could overcome therapeutic resistance to LIGHT alone. The clinical impact and antitumor immune mechanism of the combination were investigated for therapeutic potential.

## RESULTS

### Combination of intratumoral LIGHT overexpression and systemic anti–CTLA-4 effectively controls MSS CRLM development in vivo

**Fig. 1.**
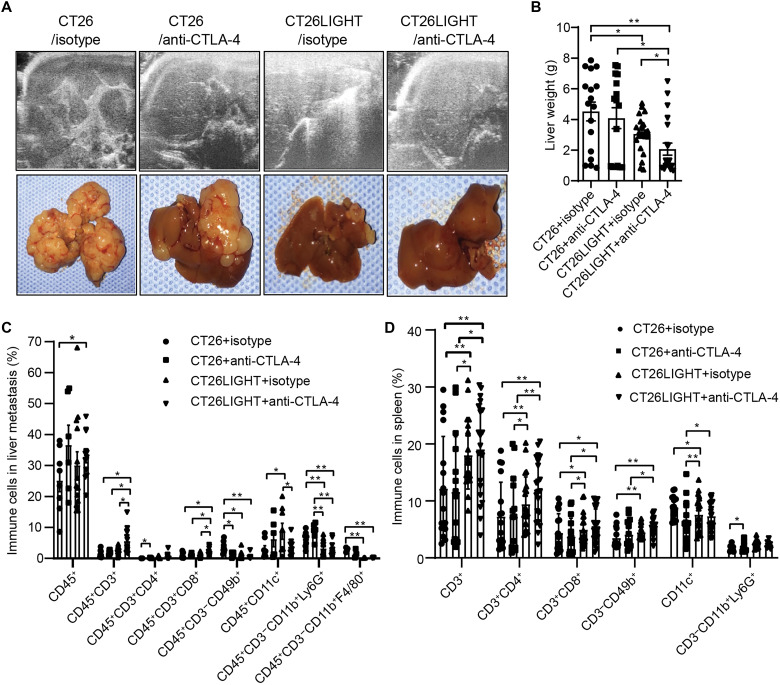
Combination treatment with LIGHT overexpression and anti–CTLA-4 effectively controls MSS CRLM development. (**A**) In vivo ultrasound images of liver with metastatic tumors (top) with associated ex vivo resected liver specimens, representative images. (**B**) Liver tumor burden is decreased in combination (LIGHT + anti–CTLA-4)–treated animals compared to control, tumor LIGHT overexpression, and anti–CTLA-4 treatment alone. We have previously shown liver weight to quantitatively correlate with metastatic liver tumor burden based on tumor area measurements in this model (*15*). Most combination-treated livers contained no liver metastases. (**C**) Tumor-infiltrating immune cell populations were assessed within liver metastases for each treatment group by flow cytometry. (**D**) Splenic immune cell populations from different treatment groups were determined with flow cytometry. Data are shown as means ± SEM. Asterisks indicate significance determined by the two-tailed unpaired *t* test between groups, *P < 0.05; **P < 0.01. *n* = 17 to 22 per group.

Animals bearing wild-type or LIGHT-overexpressing tumors were treated in a well-established immunocompetent syngeneic model of isolated CRLMs that recapitulates the human course (*15*, *16*, *18*). Critically, these CRLMs lack coding somatic mutations in *Mlh1*, *Msh2*, *Msh6*, *Pms2*, *Mlh3*, and *Msh3* and thus translationally mirror the MSS phenotype of >95% of human CRC (*19*–*21*). Animals with LIGHT-expressing CT26 CRLMs or wild-type tumors received systemic anti–CTLA-4 or isotype control. CRLMs were monitored following tumor initiation and tumor burden quantified at laparotomy. Metastatic liver tumor burden in animals that received a combination of LIGHT + anti–CTLA-4 was significantly lower than that of mice treated with LIGHT alone or anti–CTLA-4 monotherapy alone, and in this combination group, most of the livers contained no identifiable metastases (Fig. 1, A and B). Thus, tumor LIGHT expression + anti–CTLA-4 effectively controlled clinically relevant CRLM tumor development in these livers.

To understand how the combination of LIGHT + anti–CTLA-4 disrupts CRLM development, intratumoral and systemic immune responses were analyzed with flow cytometry. The combination group showed a remodeling of the TME with an increased frequency of multiple immune cell types including total CD45^+^ cells, total CD3^+^ T cells, and CD8^+^ T cells and decreased frequency of granulocytic myeloid-derived suppressor cells (G-MDSCs) and macrophages (Fig. 1C). Combination of LIGHT overexpression + systemic anti–CTLA-4 treatment also induced expansion of CD3^+^, CD4^+^, CD8^+^ T cells and natural killer (NK) cells in the spleen compared with control or anti–CTLA-4 alone (Fig. 1D). The combination treatment increased splenic CD11c^+^ dendritic cells compared to anti–CTLA-4 monotherapy (Fig. 1D). Furthermore, splenic CD8^+^ T cell activation (% CD25^+^CD8^+^), CD4^+^ T cell activation (% CD69^+^CD4^+^) and central memory, and CD8^+^ and CD4^+^ proliferation (% Ki67^+^) (fig. S1, A to H) were all increased in the combination treatment group compared to wild-type tumors. Evaluation of regulatory T cells (T_reg_ cells) in the spleen revealed that combination treatment increased T_reg_ frequency to a greater degree than all other treatment groups; however, these T_reg_ cells expressed lower levels of Granzyme B compared with anti–CTLA-4 alone–treated animals (*P* = 0.04) (fig. S1, I and J). Combined, these results support that combination treatment generates systemic and intratumoral immune activation.

Given the clinical relevance of combination checkpoint blockade with anti–PD-1 and anti–CTLA-4 in other cancers, such as melanoma, we repeated experiments with the addition of systemic anti–PD-1 antibody. In the MSS CRLM CT26 syngeneic model, adding anti–PD-1 monotherapy to LIGHT overexpression did not reduce metastatic liver tumor burden, unlike the addition of anti–CTLA-4 (fig. S2). Similarly, combining anti–PD-1 with anti–CTLA-4 did not further reduce metastatic colorectal liver tumor burden compared to anti–CTLA-4 + LIGHT (fig. S2, A and B). These results suggest that the mechanism of clinical response was specific to the combination of LIGHT + anti–CTLA-4. Immunocytes in the spleen were also evaluated after combination treatment with LIGHT overexpression, anti–PD-1, and anti–CTLA-4. Compared to LIGHT + anti–CTLA-4 treatment, neither LIGHT + anti–PD-1 nor LIGHT + anti–CTLA-4 + anti–PD-1–treated animals experienced increased populations of splenic CD3^+^CD4^+^ T cells, CD3^+^CD8^+^ T cells, CD49b^+^ NK cells, or CD11c^+^ dendritic cell populations (fig. S2C). Neither did anti–PD-1 increase the number of TILs in CRLMs compared to LIGHT + anti–CTLA-4 (fig. S2D). Thus, the mechanism of clinical response, secondary lymphoid organs, and in TILs is unique to LIGHT overexpression + anti–CTLA-4.

To evaluate the clinical findings in an additional cell line and a different animal background, MC38 syngeneic colon cancer cells were used in C57BL/6 mice CRLMs. Combination of LIGHT overexpression + systemic anti–CTLA-4 treatment revealed similar antitumor responses in this model, also demonstrating increased TILs with LIGHT overexpression (fig. S3, A to C), although it must be noted that these tumors are MSI-H and thus known to be exquisitely ICB sensitive, unlike most human CRLMs and the MSS murine CT26 CRLMs. To additionally evaluate the impact of combination treatment in human colorectal tumors, HT-29 MSS flank tumors were established in NOD scid gamma (NSG) mice reconstituted with human leukocyte antigen (HLA)–matched human peripheral blood mononuclear cells (PBMCs). In this humanized murine model, LIGHT expression was induced when tumors became palpable (fig. S3D). Animals were then treated with or without systemic anti–CTLA-4. Tumors treated with anti–CTLA-4 alone did not respond, whereas combination therapy resulted in substantial tumor regressions compared to anti–CTLA-4 treatment or LIGHT overexpression alone (fig. S3E). Combination therapy induced regression of tumors, which limited the analysis of tumor-infiltrating immune cells; however, analysis of splenic and peripheral blood immune cells demonstrated increased T cells with anti–CTLA-4 and combination LIGHT + anti–CTLA-4 treatment (fig. S3, F and G).

### Combination treatment drives TILs into liver metastases, increases infiltrating T cell activation and effector function, and decreases markers of T cell exhaustion

**Fig. 2.**
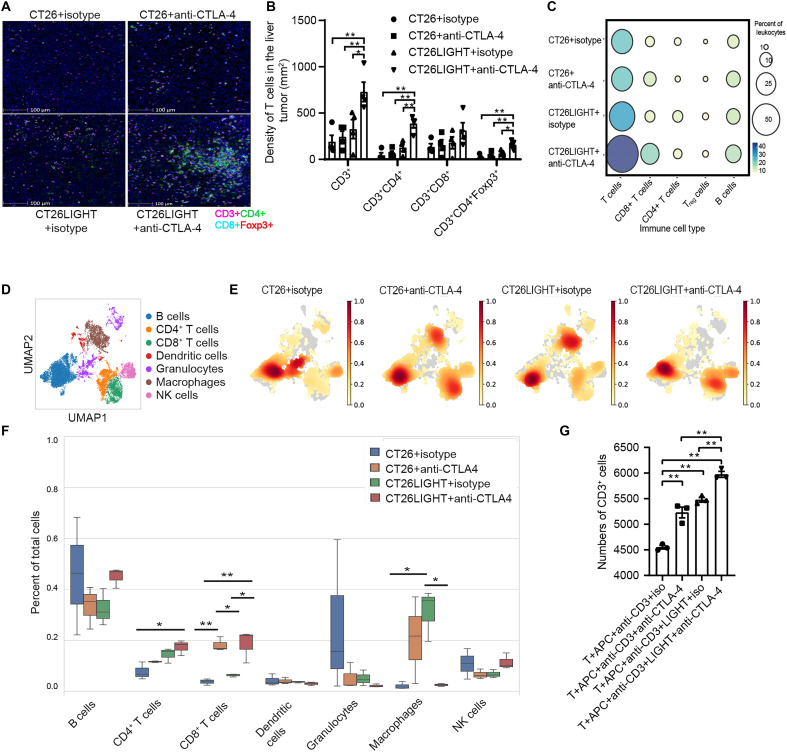
Combination treatment induces T cell proliferation and results in TME remodeling with increased TILs. (**A**) IF staining of the T cell subsets in CRLM liver tumor sections, following LIGHT induction (for mice in the CT26LIGHT groups only) and treatment with isotype control or anti–CTLA-4. Scale bars, 100 μm. (**B**) Summary of the density of T cell subsets in the liver tumor tissue from (A). Most of the CD3^+^, CD4^+^, and CD4^+^Foxp3^+^ T_reg_ cells were located in the interface of tumor tissue and normal liver tissue (*n* = 3 per group; *n* = 10 high-power fields per specimen). (**C**) Distribution of T cell subsets and B cells in hepatic leukocytes (CD45^+^) in the CRLMs from CyTOF analysis (*n* = 3 per group pooled into one sample). The size and color of the circles correspond with the frequency of each cell type. (**D**) UMAP plot of the different immune cell clusters in the CRLMs from scRNA-seq analysis (*n* = 3 per group). UMAP, Uniform Manifold Approximation and Projection. (**E**) Density heatmap of immune cell clusters is overlaid on the UMAP plot, demonstrating relative frequencies of immune cells in different treatment groups. (**F**) Frequency of immune cell clusters out of total hepatic leukocytes (CD45^+^) in CRLMs from different treatment groups analyzed through scRNA-seq. (**G**) In vitro coculture assay for CD3^+^ T cell proliferation in the presence of murine LIGHT, anti CTLA-4, or both (*n* = 3 per group). T, CD3 T cells; APC, antigen-presenting cells. All *P* values for scRNA-seq are adjusted for FDR. For the flow cytometry data, data are shown as means ± SEM. Asterisks indicating significance determined by the two-tailed unpaired *t* test between groups. *P < 0.05; **P < 0.01.

Increased trafficking of TILs into CRLMs is associated with antitumor immune responses and improved outcomes (*13*, *15*, *16*). Therefore, we assessed TIL quantity and function after combination treatment. Multicolor immunofluorescence (IF) analysis of surgically resected CRLMs revealed increased trafficking of CD3^+^ T cells into CRLMs at the tumor interface in the combination-treated tumors compared to wild-type tumors, LIGHT overexpression alone, and anti–CTLA-4 treatment alone (Fig. 2, A and B). This finding was confirmed by digesting the entire tumor, revealing that total CD3^+^ T cells were increased by flow cytometry analysis (Fig. 1C) and mass cytometry (CyTOF) (Fig. 2C) of CD45^+^ TILs. In these analyses, a combination of increased LIGHT expression plus anti–CTLA-4 resulted in maximal increases of total T cells.

We also performed single-cell RNA sequencing (scRNA-seq) of tumors, which revealed marked remodeling of the TME, showing that the combination treatment group tumors were T cell rich and relatively deplete of suppressive granulocytes and macrophages (Fig. 2, D to F). To further validate these results and investigate the mechanism behind the increased T cell proliferation found in the in vivo experiments, a T cell proliferation assay was performed with labeled purified CD3^+^ T cells ex vivo. T cell number significantly increased in the presence of both anti–CTLA-4 and recombinant LIGHT together compared to culture with isotype control, anti–CTLA-4, or LIGHT alone (Fig. 2G). These results demonstrate that the combination promotes T cell proliferation more than exposure to anti–CTLA-4 or increased LIGHT alone.

**Fig. 3.**
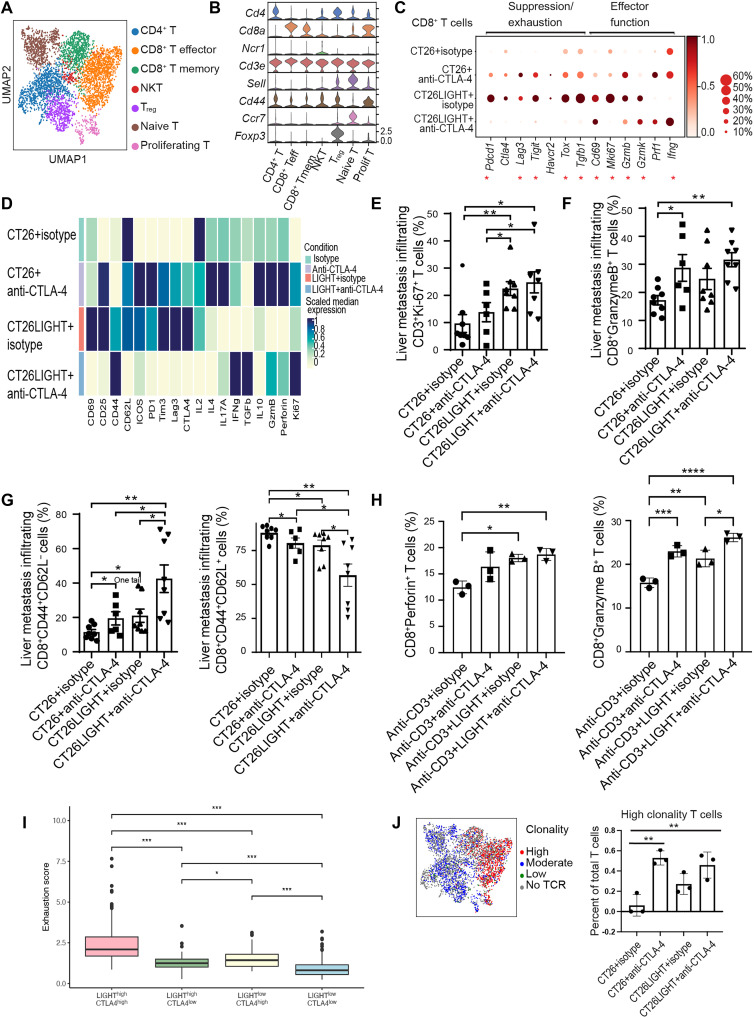
Combination treatment mitigates tumor-infiltrating CD8^+^ T cell exhaustion and promotes T cell activation, proliferation, and function. (**A** to **C**) scRNA-seq analysis of T cells from tumors (*n* = 3 per group). (A) UMAP and (B) RNA expression of marker genes used for T cell classification. CD8^+^ T_eff_, CD8^+^ T_eff_; CD8^+^ Tmem, CD8^+^ T memory; prolif T, proliferating T cell. (C) Gene expression in CD8^+^ T cells. * indicates any statistical comparisons between the CT26LIGHT + anti–CTLA-4 group and any of the other groups. (**D**) Heatmap of protein expression in CD8^+^ T cells from CyTOF analysis (*n* = 3 per group). (**E**) Percentage of Ki-67^+^ of CD3^+^ T cells via flow cytometry analysis (*n* = 6–8 per group). (**F**) Percentage of Granzyme B^+^ of CD8^+^ cells in the CRLMs from flow cytometry analysis (*n* = 6 to 8 per group). (**G**) Percentage of CD44^+^CD62L^−^ cell (T_eff_ memory) and CD44^+^CD62L^+^ cell (T central memory) out of CD8^+^ T cells via flow cytometry analysis (*n* = 6 to 8 per group). (**H**) Percentage of Perforin^+^ and Granzyme B^+^ CD8^+^ T cells after in vitro stimulation with LIGHT and anti–CTLA-4 via flow cytometry analysis. For (E) to (H), means ± SEM is shown on plots, and a two-tailed unpaired *t* test was performed between groups. (**I**) Exhaustion score is plotted for each sample in the TCGA dataset (*n* = 396 cases) stratified by *CTLA4* and *TNFSF14* expression (*n* = 148 for LIGHT^high^CTLA4^high^, *n* = 50 for LIGHT^high^CTLA4^low^, *n* = 50 for LIGHT^low^CTLA4^high^, and *n* = 148 for LIGHT^low^CTLA4^low^). Median score and interquartile range are shown. *P < 0.05; ***P < 0.005. (**J**) UMAP plot of low, moderate, and highly clonal T cells and plot of the percentage of high clonality T cells out of T cells (moderate clonality = 2 unique T cells with the same TCR; high clonality refers to >2 T cells with the same TCR). *P < 0.05; **P < 0.01; ***P < 0.001; ****P < 0.0005.

Further subclustering on infiltrating T lymphocytes using scRNA-seq focused on T cell subtypes, phenotype, and T cell receptor (TCR) repertoire including CD4^+^ T cells, CD8^+^ effector and memory cells, NK T cells, T_reg_ cells, and two clusters containing both CD4^+^ and CD8^+^ T cells (naïve cells and proliferating cells) (Fig. 3, A and B). Focusing on the CD8^+^ T cells, combination treatment up-regulated genes related to CD8^+^ T cells cytokine production and effector function compared to either LIGHT overexpression or anti–CTLA-4 treatment, particularly *Ifng* gene expression levels. (Fig. 3C). CyTOF confirmed this increased expression of interferon-γ (IFN-γ) and markers of effector function in CD8^+^ TILs and revealed an increase in CD8^+^ T cell proliferation (by Ki-67) compared with isotype or anti–CTLA-4 or LIGHT treatment alone (Fig. 3D). We also found that combination treatment increased the effector memory (CD44^+^, CD62L^−^) CD8^+^ T cell population compared with isotype control or anti–CTLA-4 or LIGHT alone (Fig. 3D). To validate the findings from scRNA-seq and CyTOF, we analyzed single-cell suspensions from CRLMs with flow cytometry. We found that there were higher frequencies of CD3^+^Ki67^+^ TILs in LIGHT overexpression alone or LIGHT + anti–CTLA-4 groups (Fig. 3E). The combination treatment group had the largest population of CD8^+^GranzymeB^+^ TILs (Fig. 3F) and effector memory CD8^+^ T cells (which expanded preferentially compared to central memory CD8^+^ T cells) compared to the control group (Fig. 3G). Using an in vitro assay, we demonstrated that T cells cultured in the presence of LIGHT and anti–CTLA-4 had a higher frequency of Granzyme B^+^ and Perforin^+^ CD8^+^ T cells than the control condition and that anti–CTLA-4 alone could also increase the frequency of Granzyme B^+^ CD8^+^ T cells as measured with flow cytometry (Fig. 3H). Collectively, from a T cell perspective, LIGHT is driving the proliferation of TILs, and anti–CTLA-4 promotes the activation.

Similar to CD8^+^ TILs, combination treatment was associated with increased *Ifng* expression and effector function compared to the other treatment groups and decreased *Tgfb1* and *Pdcd1* in CD4^+^ TILs, using scRNA-seq (fig. S4A). With CyTOF, we confirmed increased activated CD4^+^ T cell protein expression of IFN-γ, Ki-67, and Granzyme B and a greater infiltration of effector memory CD4^+^ T cells, compared with isotype control and monotherapies, although this was not significant by flow cytometry (fig. S4, B and C). We also demonstrated that CD4^+^ T cells cultured in vitro with anti–CTLA-4 had a higher frequency of Granzyme B^+^ cells and a trend toward more Perforin^+^ cells as well, as was seen in vivo (fig. S4D). Thus, there was also an increase in CD4^+^ TIL trafficking, proliferation, and activation phenotype.

To further evaluate mechanisms by which combination treatment induces increased CD4^+^ and CD8^+^ tumor infiltration, we assessed the gene expression of relevant receptors involved with lymphocyte migration into the tumor tissue. Combination treatment up-regulated expression of the homing signals *Itga4* and *Itgb7* in CD8^+^ T cells and *Itga4* in CD4^+^ T cells compared to LIGHT treatment alone, as well as increased *Itga4* in CD8^+^ T cells compared to anti–CTLA-4 monotherapy (fig. S5, A and B). IF staining for TLSs (using CD4, CD8a, and CD19) demonstrated that there were significantly more TLSs in the combination group compared to the control and either monotherapy group (fig. S5C). Further evaluation of the mechanism of action was performed with quantitative polymerase chain reaction (qPCR) of tumor tissues. Directed trafficking of immune cells, particularly T cells, to the TME with the combination of LIGHT overexpression and anti–CTLA-4 was supported by increased tumor tissue gene expression of the chemokines *Madcam1*, *Ccl21*, and *Ccl4*, which normalize tumor vasculature and induce high endothelial venules and TLSs (*22*–*24*) (fig. S5, D to F). *Ccl4* in particular is a chemoattractant for cytotoxic T cells, which is consistent with scRNA-seq results and which was increased in tumors only in the combination treatment group. We further validated the increased trafficking of T cells by quantifying the chemokines *Cxcl9*, *Cxcl10*, and *Cxcl11* in tumors, finding the highest levels in the combination group compared to controls (fig. S5, G to I). This finding supports that the tumor immune microenvironment created by combination treatment increases signals that attract activated T lymphocytes into the tumor. IFN-γ induces the expression of these chemokines involved in T cell recruitment (*25*–*27*), and accordingly, the *Ifng* gene was up-regulated with either LIGHT expression or anti–CTLA-4 compared to controls and had the highest expression in the combination compared to all other treatment groups (fig. S5J), consistent with the scRNA-seq findings. We evaluated which cell types in the TME produce these chemokines with scRNA-seq, finding that some transcripts were not detectable by this method (*28*), and for those that were (*Ccl4*, *Cxcl9*, *Cxcl10*, and *Ifng*), macrophages were a primary source; however, as our scRNA-seq experiment did not include CD45^−^ cells, we cannot fully assess the contribution of tumor and stromal cells (fig. S5K).

### Decreased lymphocyte exhaustion and increased T cell clonality

Although LIGHT expression in tumors activated CD8^+^ TILs, there was T cell exhaustion with LIGHT treatment alone. We find here that combination treatment down-regulated the exhaustion markers *Pdcd1*, *Lag3*, *Tigit*, and *Tox* compared with anti–CTLA-4 or LIGHT treatment alone (Fig. 3C). We confirmed with CyTOF that combination treatment decreased the protein expression of PD-1, CTLA-4, LAG-3, and TIM-3 in tumor-infiltrating CD8^+^ T cells compared with anti–CTLA-4 or LIGHT treatment alone (Fig. 3D). We also found a similar effect of CTLA-4 up-regulation (CD8^+^ T cells: not significant; CD4^+^ T cells: *P* = 0.05) and decreased CD8^+^ T cell proliferation and IFN-γ production in vitro when LIGHT alone is cultured with stimulated T cells compared to LIGHT and anti–CTLA-4 (fig. S6). Thus, combination treatment functions, at least in part, through mitigating tumor-infiltrating CD8^+^ T cell exhaustion and further enhancing effector functions including IFN-γ production.

We next investigated this finding in 396 human colon adenocarcinomas (*20*), stratified samples by expression of LIGHT (*TNFSF14*) and CTLA-4 and applied a T cell exhaustion gene signature. Tumors with high LIGHT had higher exhaustion scores than LIGHT^low^ tumors, suggesting that, although LIGHT can induce and recruit effector T cells, they are also subject to exhaustion. Tumors with high LIGHT and high CTLA-4 expression had higher exhaustion scores than LIGHT^high^CTLA-4^low^, which corresponds with our data showing that T cell exhaustion is mitigated in the LIGHT-treated tumors with the addition of anti–CTLA-4 (Fig. 3I).

Furthermore, as antigen-specific T cell clonal expansion may indicate tumor antigen specificity, we investigated the TCR repertoire and found that the addition of anti–CTLA-4 in the control or LIGHT-overexpressing tumors was associated with increasing T cell clonality (Fig. 3J). LIGHT overexpression alone could not significantly increase clonality. Combination treatment induced multiple T cell clones to expand beyond the known tumor-associated antigen AH1 compared with the monotherapy groups (Table 1).

**Table 1.** Ten most frequent T cell clones in each treatment group. TCR sequencing was performed along with scRNA-seq of CRLMs, and the top 10 most frequent β chain sequences are shown for each treatment group (*n* = 3 per group). The total number of T cells with each sequence is noted, along with the total frequency of these top 10 clones out of the total TCRs retrieved and the percent of the top 10 that are sequences known to be specific for AH1.

Group	CDR3β	Frequency (*n*)	% of total T cell clones	% AH1-specific clones
wtCT26+isotype	CTCSGTGSYAEQFF	7		
	CTCSAGQKNERLFF	2		
	CAWRAWGSAETLYF	2		
	CASSPGTGGERLFF	2		
	CASGPGGRDTQYF	2		
	CTCSAETGVQDTQYF	2		
	CTCSGHNERLFF	2		
	CASSDAAGGTERLFF	2		
	CTCSAGLGGAETLYF	2		
	CASQGNTGQLYF	2	17.6	0
wtCT26+anti–CTLA-4	CASSSRTGGYAEQFF	38		
	CASSLEINQDTQYF	20		
	CAWSPGTGGNERLFF	19		
	CASSTRTGGYAEQFF	18		
	CASSSRQGGYAEQFF	17		
	CASREDRGGQNTLYF	16		
	CASSQGTSANTEVFF	12		
	CASSIKLGGYAEQFF	10		
	CAWSLTGGGYAEQFF	9		
	CASSLRTGGYAEQFF	9	46.28	23.42
CT26LIGHT+isotype	CASSIKTGGYAEQFF	20		
	CASSTRTGGYAEQFF	16		
	CASSLTGGAETLYF	12		
	CASSPQGAREQYF	8		
	CASSIKTGGFAEQFF	8		
	CASSSGTANTEVFF	8		
	CASSLRTGGYAEQFF	7		
	CASSPQGAREQYF	7		
	CASSAGGSDYTF	6		
	CASSLTGGAETLYF	6	66.22	38.51
CT26LIGHT+anti–CTLA-4	CASSWTGGAETLYF	53		
	CASSDAGGQDTQYF	34		
	CASSAFYEQYF	14		
	CASGWDREVFF	13		
	CASSGGAAEQFF	11		
	CAWSPGDNTEVFF	11		
	CASSLVGGREQYF	10		
	CASGDLGENYAEQF	10		
	CASGGAGGAETLYF	10		
	CASSSDRAGYAEQFF	9	55.73	0

### T_reg_ cells: Combination treatment inhibits T_reg_ function and increases the ratio of intratumoral effector T cells to T_reg_ in CRLMs

**Fig. 4.**
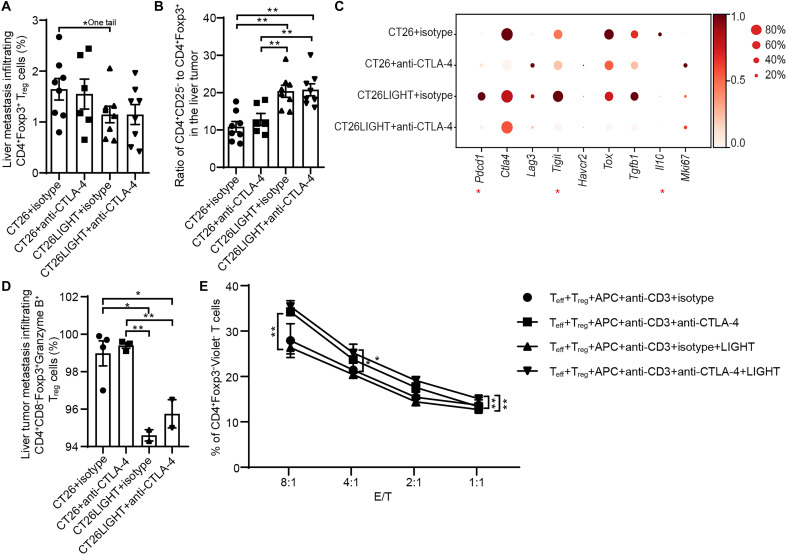
Combination treatment inhibits T_reg_ function and increases the ratio of T_eff_ to T_reg_ in CRLMs. (**A**) Frequency of T_reg_ cell in the CRLMs assessed via flow cytometry analysis. (**B**) Ratio of CD4^+^CD25^−^ (T_eff_) to T_reg_ cell in the CRLMs from the different treatment groups assessed via flow cytometry analysis (*n* = 6 to 8 per group). (**C**) Expression levels of genes related to proliferation, exhaustion, and function of T_reg_ cells in CRLMs from scRNA-seq analysis (*n* = 3 per group). In the expression plot, * indicates any statistical comparisons between the CT26LIGHT + anti–CTLA-4 group and any of the other groups. (**D**) Percentage of tumor-infiltrating Foxp3^+^ T_reg_ cells in CRLMs that are Granzyme B^+^ assessed via flow cytometry analysis (*n* = 3 to 5 per group). (**E**) T_eff_ (CD4^+^CD25^−^ T_eff_ cells) were cocultured in vitro in the presence of murine LIGHT and/or anti–CTLA-4. T_eff_ proliferation was measured with flow cytometry as a correlate of T_reg_-induced suppression (*n* = 3 per group). Data are shown as means ± SEM. Asterisks indicating significance determined by the two-tailed unpaired *t* test between groups, *P < 0.05; **P < 0.01.

The proportion of T_reg_ in CRLM tumors does not decrease substantially with combination treatment (Fig. 4A), and when evaluated by IF staining at the tumor interface, recruited T_reg_ density may actually increase (Fig. 2, A and B). Although combination treatment was associated with an increase in all CD4^+^ T cell subsets compared to other treatment groups, there was an increase in the ratio of effector T (T_eff_; CD4^+^CD25^−^) to T_reg_ (CD4^+^Foxp3^+^) in the LIGHT-overexpressing and combination-treated CRLMs compared with isotype control or anti–CTLA-4 treatment alone (Fig. 4B). Furthermore, combination treatment down-regulated expression of *Pdcd1* and *Tigit* in tumor-infiltrating T_reg_ compared with LIGHT treatment alone and decreased *Il10* compared to the control group, implying that these T_reg_ cells have decreased suppressive function in the combination-treated tumors (Fig. 4C). Further evaluation by flow cytometry revealed down-regulated Granzyme B expression in tumor-infiltrating T_reg_ cells compared with isotype control or anti–CTLA-4 treatment alone (Fig. 4D). To further investigate T_reg_ cell function, T_eff_ (CD4^+^CD25^−^) and T_reg_ were cocultured in a suppression assay at different ratios. Anti–CTLA-4 plus LIGHT treatment reduced T_reg_ suppressive function compared with LIGHT alone or isotype when T_eff_:T_reg_ were cocultured at 1:1 (Fig. 4E).

### Myeloid cells: Combination treatment decreases tumor-infiltrating macrophages and MDSCs and promotes dendritic cell maturation

**Fig. 5.**
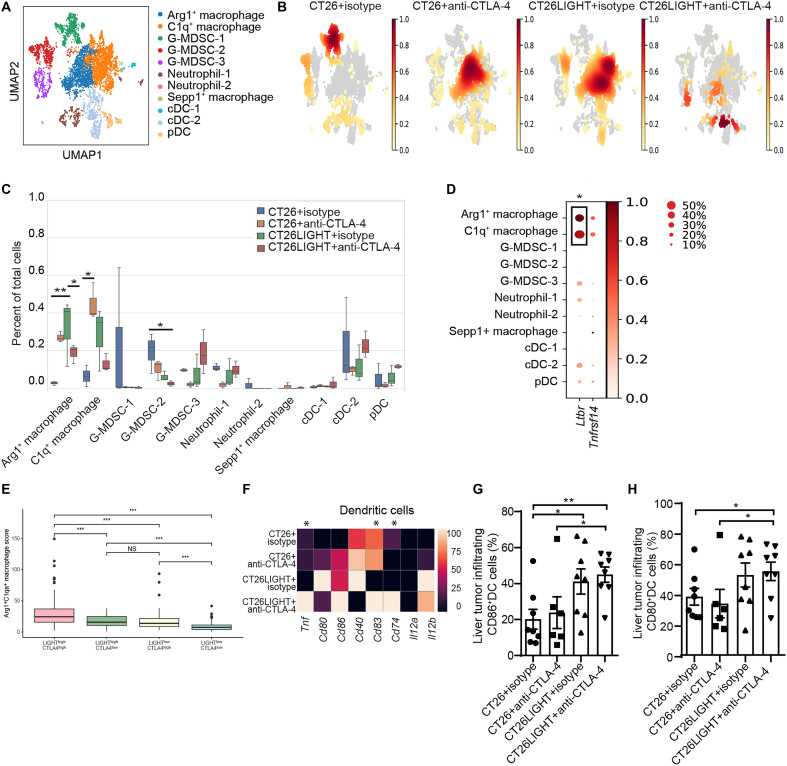
Combination treatment restricts tumor-infiltrating macrophages and MDSCs and promotes cDC maturation. (**A**) UMAP plot of myeloid cell clusters in CRLMs from scRNA-seq analysis (*n* = 3 per group). (**B**) Density heatmap of myeloid cell clusters is overlaid on the UMAP plot, demonstrating relative frequencies of myeloid cells in different treatment groups. (**C**) Frequency of myeloid cell clusters in CRLMs from different treatment groups analyzed through scRNA-seq. (**D**) Expression levels of *Ltbr* and *Tnfrsf14* in myeloid subpopulations from scRNA-seq analysis. In the expression plot, * indicates any statistical comparisons between the macrophages and all other myeloid cell types. (**E**) Arg1^+^/C1q^+^ macrophage score is plotted for each sample in the TCGA dataset (*n* = 396 cases) stratified by *CTLA4* and *TNFSF14* expression (*n* = 148 for LIGHT^high^CTLA4^high^, *n* = 50 for LIGHT^high^CTLA4^low^, *n* = 50 for LIGHT^low^CTLA4^high^, and *n* = 148 for LIGHT^low^CTLA4^low^). Median score and interquartile range are shown. ***P < 0.005; NS, not significant. (**F**) Heatmap of gene expression associated with DC maturation and function through scRNA-seq. In the expression plot, * indicates any statistical comparisons between the CT26LIGHT + anti–CTLA-4 group and any of the other groups. (**G** and **H**) CD86 and CD80 expression on DC in the CRLMs from different treatment groups assesses via flow cytometry analysis (*n* = 6 to 8 per group). Data are shown as means ± SEM. Two-tailed unpaired *t* test between groups. Asterisks indicating significance determined by the two-tailed unpaired *t* test between groups, *P < 0.05; **P < 0.01; ***P < 0.001.

To further investigate myeloid cells within the TME, we analyzed several MDSC, macrophage, and dendritic cell populations at the single-cell level. Without any treatment, CRLMs contain a large subpopulation of *Ly6g*^+^*Mmp9*^+^ G-MDSCs (G-MDSC-1; Fig. 5, A and B). This population became barely detectable with LIGHT overexpression, anti–CTLA-4 therapy, and combination treatment. Untreated liver metastases in general contained very few macrophages, and both LIGHT overexpression and anti–CTLA-4 treatment substantially increased this population. However, combination treatment returned these populations to near baseline (Fig. 5, A to C). To further differentiate the functional macrophages within this group, they were classified (fig. S7) by gene expression of *Arg1*, *C1qa*, *Csf1r*, *Vegfa*, *Trem2*, *Entpd1*, *Sirpa*, *Tgfb1*, and *Cd274,* indicating a more M2-like phenotype (*29*–*34*). The tumor-promoting *Arg1*^+^ and *C1qa*^+^ macrophages had higher expression of the LIGHT receptor gene *Ltbr,* but not *Tnfrsf14*, than other myeloid cell types (Fig. 5D), which likely contributed to the modulation of these macrophages with LIGHT overexpression. These suppressive macrophages markedly increased with monotherapies, however, were excluded from the tumor only with combination of LIGHT overexpression and anti–CTLA-4 treatment. There was also a decreased frequency of the *Mmp9*^+^*Vegfa*^+^ G-MDSC-2 with combination therapy (Fig. 5C and fig. S7). In in vitro coculture experiments, we found that the combination therapy resulted in the lowest frequency of G-MDSCs (*P* = 0.0015 compared to control condition), C1qa^+^ (*P* = 0.0052), and Arg1^+^ macrophages (*P* = 0.0019). The human correlate of this finding was evaluated using the CRC TCGA (The Cancer Genome Atlas) data in which we were able to demonstrate that, in tumors with high LIGHT and high CTLA-4 expression, there was increased suppressive myeloid cell score compared to tumors with high LIGHT and low CTLA-4 expression, demonstrating that the combination of LIGHT overexpression and anti–CTLA-4 may be able to overcome myeloid suppression in tumors from patients (Fig. 5E). As with the mouse data, the addition of LIGHT alone could increase the suppressive macrophages; therefore, other means of immune activation (i.e., CTLA-4 blockade) may be needed to overcome the increase in suppressive macrophages in the TME.

There were no significant changes in the frequency of conventional dendritic cells (cDCs) in the tumors by the treatment group; however, there was evidence of increased activation of cDCs in the combination group, including higher expression of *Tnf* and *Cd83* compared to the LIGHT alone group and higher expression of *Cd74* than the control and anti–CTLA-4 groups (Fig. 5F). There was a higher frequency of CD86^+^ and CD80^+^ cDCs in the combination compared to anti–CTLA-4 alone and the control groups with flow cytometry, demonstrating evidence of cDC activation (Fig. 5, G and H). Collectively, combination treatment promotes tumor-infiltrating DC activation and maturation while decreasing certain populations of suppressive macrophages and MDSCs.

## DISCUSSION

Immunostimulatory and proinflammatory cytokines have been used to treat patients with cancer for decades, including early trials using interleukin-2 (IL-2) for patients with metastatic cancers (*3*, *35*). Similarly, when ICB was initiated in patients with melanoma and renal cell cancer, clinical responses were equally provocative. Their use, however, has been severely limited in MSS tumors, like CRC, where clinical responses have been negligible (*4*, *5*). Nonetheless, strategies to enhance cytotoxic T lymphocyte function remain a viable cancer treatment approach, and TNFSF14/LIGHT is an immunostimulatory signal that can activate antitumor immune responses in the TME (*36*, *37*). We previously evaluated the tumor immune microenvironment of resected CRLMs and determined that tumor recurrence and patient survival were enhanced when LIGHT expression was increased in the tumors. What was not previously fully understood is how LIGHT overexpression acts on the TME of MSS (“cold”) CRLMs and affects the balance of tumor-promoting and tumor-suppressive elements nor how to overcome this. Furthermore, to have potential for patient treatment, complete tumor regressions are necessary and had not yet been achieved in MSS CRLMs. On the basis of CTLA-4^+^ TILs specifically trafficking to CRLMs, a combination of LIGHT with anti–CTLA-4 ICB could provide this balance. Thus, the combination strategy was evaluated and the mechanism of clinical response was determined.

We demonstrate that combination treatment with LIGHT expression and anti–CTLA-4 effectively controls MSS CRLMs development in vivo, which justifies evaluation of the mechanism of action and clinical potential. This could not be accomplished with either monotherapy alone in the liver, which is well accepted to be an immune-privileged niche (*38*), or with anti–PD-1 treatment. Further evaluation of the treatment response revealed that combination treatment generated T cell activation, proliferation, and memory in secondary lymphoid organs. That effector memory CD44^+^CD62L^−^CD8^+^ cells were also increased in the combination-treated animals reinforces the impact this strategy can have on recall and durability of response, e.g., RFS. The combination treatment also drives migration of TILs into the liver metastases, and these T cells demonstrate increased activation and effector function, and, critically, decreased markers of exhaustion compared to LIGHT overexpression alone. Enhanced trafficking of T cells into the liver metastases and the formation of TLSs within the tumors were supported by increased expression of genes known to normalize the tumor vasculature and induce high endothelial venules and TLSs seen only with the combination of LIGHT and anti–CTLA-4 (*22*–*24*). This may account for differences between T cell numbers within the spleen and liver metastases within the combination group. Collectively, from a T cell perspective, LIGHT is driving the proliferation of TILs as well as some suppressive elements, and anti–CTLA-4 promotes the activation. Our data suggest that the primary function of CTLA-4 blockade in CRLMs is to activate T cells rather than to reduce the T_reg_ population in tumors, although we observed decreased T_reg_ function and ratios with combination treatment. This differs from its proposed role in some “hot” immunosensitive tumors, where it primarily targets and depletes T_reg_ cells. Further evaluation of CTLA-4 blockade’s impact on T cell priming in the spleen may provide greater insight as the observed effects on exhaustion and suppressive myeloid populations suggest a more complex mechanism underlying the complementary effects of the combination treatment. One could speculate that the earlier mechanism of action of anti–CTLA-4 in T cell priming compared to anti–PD-1 is necessary to overcome T cell exhaustion (*39*).

We show that both TME LIGHT overexpression and anti–CTLA-4 treatment alone can drive cytotoxic T lymphocytes to exhaustion, a limitation to either approach. However, when given together, they can decrease markers of exhaustion, representing not an incremental but a considerable step forward for therapeutic potential compared to previous studies. This is further supported by evidence we show in resected human colorectal tumors of lower T cell exhaustion scores in the LIGHT^high^CTLA-4^low^ population as a surrogate of LIGHT overexpression and CTLA-4 blockade. T cell exhaustion is multifactorial, and our data support that, although PD-1 is a useful marker, it must be interpreted in the context of a much larger exhaustion signature, which we show to include other markers including CTLA-4, TIGIT, Havcr2 (TIM-3), Tox, and LAG-3. Furthermore, the contribution of DC activation cannot by underestimated as a mechanism for the T cell activation and that CD80 and CD86 are only up-regulated with intratumoral LIGHT and when combined with anti–CTLA-4 is particularly relevant.

From the perspective of managing immune suppressive signals in the TME, it was demonstrated that combination treatment inhibits T_reg_ function and increases the ratio of T_eff_ cells to T_reg_ in CRLMs. In the spleen, combination treatment increased T_reg_ compared to all other treatment groups; however, these T_reg_ expressed lower levels of Granzyme B compared with isotype control or anti–CTLA-4 alone–treated animals, implying decreased ability to suppress tumor clearance and less suppressive T_reg_ cells (*40*). Similarly, populations of MDSCs present in untreated tumors are significantly depleted only in the combination-treated animals. Arg1^+^ and C1q^+^ macrophages, known to dampen T cell activation, are both associated with tumor progression (*31*, *41*), and although they are not abundant in untreated tumors, they markedly increase with both LIGHT overexpression or anti–CTLA-4 treatment. This highlights the challenge of immunostimulation where both tumor suppressing and inhibiting immunocytes will be activated. Both the Arg1^+^ and C1q^+^ M2-polarized macrophage populations express high levels of the LIGHT receptor gene *Ltbr*, which may account for their proliferation upon LIGHT engagement or ICB (*42*, *43*). However, combination treatment effectively depletes these macrophages. The decrease in tumor-associated macrophages with the combination was supported by analysis of patient tumors in which the LIGHT^high^CTLA-4^low^ population had lower *ARG1*/*C1QA* macrophage scores than LIGHT^high^CTLA-4^high^ tumors. Many current immunotherapies are limited in their antitumor efficacy by stimulation of or an inability to suppress tumor-associated macrophages, and we show the combination of LIGHT + anti–CTLA-4 to address this concern.

In addition to promoting DC maturation and CD8^+^ T cell homing to CRLMs, using both LIGHT overexpression and anti–CTLA-4 increased the spectrum of recognized T cell antigens. T cell clones responding to tumor antigens are unique in the combination-treated group and less dependent on the known tumor-associated antigen AH1, indicating expansion of a pool of T cells that are activated uniquely with the combination treatment. This is notable in light of recent studies demonstrating the success of cancer vaccines inducing neoantigen-specific responses, which correlate with clinical responses (*44*, *45*).

There are limitations to the study. As patients with MSS CRC and CRLMs are not being treated now with anti–CTLA-4 therapy alone or with tumor-directed LIGHT, experiments were performed in a clinically relevant and well-established model of CRLMs. Compared to orthotopic injection, this model is more physiologically similar to patients, with metastasis via the portal vein. A human-derived tumor CRLM model in a humanized mouse is being tested but has a limited myeloid cell compartment, which we have shown here to be a critical component of the TME. Spontaneously developed tumors in genetically modified mouse models can mimic human CRC and colitis-related carcinogenesis, but the liver metastatic incidence and severity of tumors are challenging to control or to manipulate for LIGHT overexpression. A subcutaneous tumor mouse model is easy to establish, and we have performed these experiments previously; however, the immune response in the TME is markedly different in liver metastases (*46*–*49*), thus we performed these experiments in the CRLM model that reflects this systemic suppression of antitumor immunity (*21*, *38*, *50*). This model is clinically relevant; however, as we are intervening with immunotherapy at the time that the liver metastases are being established, it is more challenging to determine effects on established tumors such as with subcutaneous models (*51*).

Furthermore, there are limited established syngeneic murine colon cancer cell lines in which to test the hypothesis and study mechanism of action. CT26 cancer cells have predominantly C > T/G > A single nucleotide variant (SNV) (*19*), similar to primary human nonhypermutated CRC tumors (*20*). The mutational signatures are DNA mismatch repair proficient (pMMR/MSS) and lack coding somatic mutations in *Mlh1*, *Msh2*, *Msh6*, *Pms2*, *Mlh3*, and *Msh3* (*19*–*21*). Together, these tumors thus were the most translatable to human clinical care. MC38 murine colon cancer cells are another option; however, they contain C > A/G > T SNVs similar to human hypermutated CRC tumors containing POLE mutations (*52*, *53*), are known to be ICB sensitive and are DNA mismatch repair (MMR) deficient (dMMR/MSI-H) (*21*, *54*). Regardless, it was important to test clinical responses in another syngeneic model in vivo (reported in the Supplementary Materials using MC38); however, any mechanistic studies would be limited and not clinically relevant to the patient population of interest. On the basis of our observations in resected CRLMs from patients, where >95% are MSS, we performed the experiments with CT26 CRC cells, which have a similar phenotype and in which liver metastases specifically been demonstrated to reflect the environment of resected tumor samples from patients (*15*, *16*). A further limitation of the model is that, given the responsiveness of MC38 tumors to anti–CTLA-4 therapy and their failure to form progressive tumors under this treatment, evaluation of immune infiltration is limited. Assessing checkpoint blockade at alternative time points may allow for investigation of immune infiltration dynamics throughout treatment. One strength, as well as a limitation, of our experiments is that we used a variety of methods (scRNA-seq, CyTOF, and flow cytometry) to investigate mechanisms, resulting in some discrepancies in mRNA and protein levels of some cytokines such as Granzyme B and TGFβ (transforming growth factor–β). This may be related to known posttranscriptional and posttranslational modifications (*55*–*59*); however, overall, our results point to a consistent increase in effector cytokines in the combination group. Furthermore, the mechanistic observations reported here warrant additional validation potentially supported by conditional knockouts or depletion of cell types.

Given the success of ICB in treating patients with various tumor biologies, there is a great interest in expanding their use and in examining new combinations. To date, however, with few exceptions in solid tumors, responses remain limited to a minority subset of patients and complete responses are rare. This is particularly true in the setting of liver metastases where even tumors that are traditionally ICB sensitive become insensitive when liver metastases are present (*38*), driven by the immunosuppressive liver microenvironment (*60*). Multiple combinations of chemotherapy, molecular targeted therapy, and ICB have been used in these patients, yet responses remain the lowest when liver metastases are present (*9*). Successful strategies may need to first recruit and support TILs in the liver tumor TME, which we demonstrated can be accomplished with LIGHT overexpression. We then showed that this can be combined with anti–CTLA-4 to overcome other immunosuppressive barriers in a clinically relevant approach. A particularly important and translational consequence of this strategy is that tumor responsive T cell clonality increases. Given the low tumor mutational burden of MSS CRLMs, and of most tumors, ICB response without this strategy is unlikely. Challenges and opportunities for current practice will be to use immunomodulatory combinations that have demonstrated a rational mechanism at a biological level, particularly in liver metastases, and to successfully deliver these therapies locally. LIGHT has been feasibly delivered to tumors through direct tumor injection, transfection, as viral cargo, via mesenchymal stem cells and TILs, and using lipid nanoparticles, as demonstrated in our studies and others across multiple tumor types (*15*, *61*–*66*). Because anti–CTLA-4 is already being delivered systemically in patients (*67*–*70*), this combination therapy is both translational and highly feasible.

## MATERIALS AND METHODS

### Experimental design

The objective of the study was to determine whether combining LIGHT expression with anti–CTLA-4 could control liver metastases in a clinically relevant model of MSS CRC by enhancing T cell activation, overcoming exhaustion, and reshaping the TME. Immune cell frequencies and phenotypes were measured by flow cytometry, mass cytometry, IF, and scRNA-seq. All experimental mouse protocols followed NIH guidelines and were approved by the Institutional Animal Care and Use Committees at the University of Illinois at Chicago (ACC#:19-170) and University of California San Francisco (#AN205618).

### Mice, mouse diet, and cell lines

BALB/c, C57BL/6, and NSG mice were purchased from Charles River Laboratory (Wilmington, MA, USA). A doxycycline diet (TD.08434) was purchased from Envigo (Madison, WI, USA) and used for induced LIGHT expression as previously described (*15*). CT26 and HT-29 colorectal carcinoma cell lines (American Type Culture Collection, Manassas, VA, USA) were grown in RPMI 1640 culture medium (Life Technology, Grand Island, NY, USA) supplemented with 10% fetal bovine serum (Invitrogen, Waltham, MA, USA). MC38 murine colon carcinoma cell line (National Cancer Institute/NIH) was grown in Dulbecco’s modified Eagle’s medium (DMEM) culture medium (Life Technologies, Grand Island, NY, USA) supplemented with 10% fetal bovine serum. CT26LIGHT and CT26LIGHT^i^ (inducible LIGHT) cell lines were generated as previously described (*15*).

### Antibodies, enzymes, and reagents

Anti–CTLA-4 (9H10), anti–CTLA-4 isotype (Syrian hamster IgG), anti–PD-1 (RMP-1-14), and anti–PD-1 isotype (2A3) were used (Bio X Cell, Lebanon, NH). For the HT-29 experiments, we used anti–hCTLA-4 (ipilimumab biosimilar SIM 0004) and human IgG1 isotype control (Bio X Cell, BP0297, Lebanon, NH, USA). LTβR-Ig was kindly provided by Y. Fu (University of Chicago, Chicago, USA). Collagenase IV and DNase I were purchased from Sigma-Aldrich (St. Louis, MO, USA). For the in vitro experiments, we used murine anti-CD3 (Bio X Cell, #BE0001-1), murine recombinant LIGHT (R&D Systems, Minneapolis, MN, USA, #1794-LT-025/CF), and Syrian hamster isotype control (Bio X Cell, #BE0087) or murine anti–CTLA-4 (Bio X Cell, #BE0131). All conjugated antibodies for CyTOF were purchased from Fluidigm Corporation (South San Francisco, CA, USA) (table S1). Flow cytometry antibody clones and source are listed in table S2. The scRNA-seq kit and reagents were purchased from 10x Genomics (Pleasanton, CA, USA). PBMCs were purchased from Lonza Biosciences (Cambridge, MA, USA).

### Construction and generation of adenoviruses

Adenoviruses were constructed as previously described (*71*). In brief, the cDNA of murine LIGHT was cloned downstream of the cytomegalovirus (CMV) promoter to create an adenovirus expressing murine TNFSF14 (Ad-LIGHT^onco^). Adenovirus replication is controlled by a TERTp located upstream of E1A. Adenovirus without a transgene (Ad-Null^onco^) was used as control vector. Adenoviral vectors were grown in human embryonic kidney (HEK) 293 cells and purified by double CsCl_2_ gradient centrifugation (*72*). Viral particle (VP) numbers were determined by measuring the optical density at 260 nm (OD_260_) of SDS-treated purified adenoviral solutions. Titers were determined by the formula: Particles/ml = (OD_260_) x (dilution factor) x (1.1 × 10^12^). LIGHT expression in MC38 cells transfected to constitutively express the human Coxsackievirus and Adenovirus Receptor (hCAR) was confirmed by flow cytometry.

### In vitro assays

For the T cell proliferation assay in vitro, purified CD3^+^ T cells (2 × 10^5^) were isolated with a magnetic isolation kit (Miltenyi Biotech, Waltham, MA, USA), labeled with trace violet and cultured with 2 × 10^5^ of T cell–depleted irradiated splenocytes. Anti-CD3 at 0.5 μg/ml with isotype control, anti–CTLA-4 at 50 μg/ml, and/or recombinant murine LIGHT at 1 μg/ml were added to the culture. For the cytokine production assay, T cells were first incubated at 37°C, 5% CO_2_, for 48 hours and then restimulated with phorbol 12-myristate 13-acetate (PMA) (50 ng/ml) and ionomycin (1 μg/ml) (Thermo Fisher Scientific, Waltham, MA, USA) in the presence of protein transport inhibitor cocktail (Invitrogen) for 5 hours, and the T cells were collected and stained for flow cytometry. For the T_reg_ suppression assay in vitro, purified T_eff_ cells (CD4^+^CD25^−^) (1 × 10^5^) were labeled with trace violet and cultured with 1 × 10^5^ T cell depleted irradiated splenocytes. T_reg_ (CD4^+^CD25^+^) were seeded at concentrations from 1 × 10^5^ to 0.125 × 10^5^ into a 96-well plate. Anti-CD3 at 0.5 μg/ml with isotype control, anti–CTLA-4 at 50 μg/ml, and/or recombinant murine LIGHT at 1 μg/ml were added. Cells were cultured in an incubator at 37°C with 5% CO_2_ for 72 hours before analysis. For the T cell exhaustion analysis, 2 × 10^5^ T cells were plated per well in medium containing IL-2 (10 ng/ml). T cells were stimulated with plate-bound anti-CD3 (2 μg/ml) and anti-CD28 (2 μg/ml) (or left unstimulated as a control) and restimulated every 48 hours. For conditions with recombinant LIGHT, plate-bound murine LIGHT was used at a concentration of 1 μg/ml. On day 8, cells were harvested, counted, and analyzed via flow cytometry. For coculture monocyte assays, we isolated bone marrow–derived monocytes from the tibias and femurs of 6- to 8-week-old BALB/c mice and purified them with a magnetic isolation kit (Miltenyi Biotech). The purified monocytes and T cells were seeded in U-bottom 96-well plates coated with murine anti-CD3 and recombinant LIGHT at 1 μg/ml in the presence of isotype control or anti–CTLA-4 at 50 μg/ml (monocytes:T cells = 5 × 10^4^:1 × 10^5^ per well). The cells were incubated at 37°C, 5% CO_2_, for 72 hours, harvested, and stained for flow cytometry. All the experiments were performed in triplicate.

### Cell staining and flow cytometry analysis

A total of 1 × 10^6^ cells were harvested and suspended in 100 μl of phosphate-buffered saline (PBS) containing 0.5% fetal bovine serum, incubated at 4°C for 30 min after adding fluorochrome-conjugated antibodies, and then analyzed after washes using a BD LSRFortessa analyzer (San Jose, CA, USA). Events were collected and analyzed using FlowJo software V10.8.1 (Treestar Incorporated, Ashland, OR, USA).

### Mouse models of CRLMs

Isolated CRLMs were generated using a well-established translational preclinical model as previously described (*16*). In brief, the spleen is carefully divided and tumor cells are inoculated into the inferior pole to replicate metastases to the liver via the portal vein. The inferior pole is then surgically removed, retaining the superior pole for immune function and evaluation, and ensuring no local tumor growth or carcinomatosis from the inoculated hemi spleen. In experiments evaluating anti–CTLA-4 or anti–PD-1 in combination with LIGHT overexpression, mice were randomized into groups of five to seven mice each. The mice were treated with four intraperitoneal doses (200 μg each) of anti–CTLA-4, anti–PD-1, both or isotype control on days 4, 7, 10, and 13 after tumor cell inoculation. To induce LIGHT expression in CT26 CRLMs, mice were fed a doxycycline-enriched diet beginning 3 days before cell injection and maintained daily thereafter. Control mice with CT26 tumors were fed a normal diet. LIGHT expression was induced in MC38 CRLMs by administration of four doses of Ad-LIGHT^onco^ (7.5 × 10^10^ viral particles (VPs) per mouse, intravenously), and animals received isotype control or anti–CTLA-4 (200 μg/mouse) intraperitoneally on days 3, 6, 9, and 12 after tumor cell inoculation (*n* = 6 each). Control animals received Ad-null^onco^. Tumor growth was monitored using a Vevo2100 Ultrasound Machine (Visual Sonics Inc., Toronto, Ontario, Canada). In the CRLM model, humane endpoints were (i) greater than 20% net body weight loss or total body weight gain of 15% or more, (ii) severe abdominal distention, and (iii) prominent bones as a result of muscle mass loss. For subcutaneous injections, humane endpoints were (i) tumor size exceeding 15% of the preoperative body weight, (ii) largest tumor diameter of 2.0 cm, (iii) greater than 20% body weight loss (minus tumor weight) or total body weight gain of 15% or more, (iv) ulcerated or infected wounds, and (v) prominent bones as a result of muscle mass loss. We have previously confirmed LIGHT expression in tumors with these well-established protocols and demonstrated liver tumor metastatic burden to correlate with liver weight in this model (*15*). Liver tumor cells and splenocytes were isolated as previously described (*15*). For the HT-29 cell line experiments, 6-week-old female NSG mice were inoculated subcutaneously with 5 × 10^6^ HT-29hLIGHTi cells in 100 μl of PBS in the right flank on day 0. On the same day, mice were administered HLA partially matched human PBMCs to HT-29 cells (catalog no. CC2703, Lonza Rockland Inc., Rockland, ME, USA) via the retro-orbital vein at a dose of 15 × 10^6^ cells in 100 μl of PBS per mouse. Mice in the LIGHT group were fed a doxycycline diet when the tumor volume reached 150 to 200 mm^3^ (day 10), whereas the control group mice were fed a normal diet after being randomized according to tumor volume. Mice were administered four doses of PBS, isotype control (200 μg in 100 μl of PBS), and anti–CTLA-4 (200 μg in 100 μl of PBS), respectively, at 3-day intervals starting on day 13 after tumor cell inoculation. The mice were euthanized 24 hours after the last treatment.

### IF staining of liver tumors

Tumor tissues were fixed in 10% formalin for 48 hours, processed in an ASP300S automated tissue processor (Leica Biosystems, Buffalo Grove, IL, USA) using the standard overnight processing protocol, and embedded into paraffin blocks. Tissue was sectioned at 5 μm, and sections were deparaffinized and stained on a BOND RX autostainer (Leica Biosystems) with a multiplex panel consisting of CD8α antibody (1:200, clone D4W2Z, Cell Signaling Technology, Danvers, MA, USA), CD3 (1:200, clone SP7, Abcam, Cambridge, UK), FoxP3 (1:100, clone D6O8R, Cell Signaling Technology), and CD4 (1:100, clone D7D2Z, Cell Signaling Technology). 4′,6-Diamidino-2-phenylindole (DAPI) staining was performed with reagents from the Opal 7-color Automation IHC Kit (Akoya Biosciences, #NEL821001KT, Menlo Park, CA) and Opal Polymer anti-Rabbit HRP Secondary Antibody Kit (Akoya Biosciences, #ARR1001KT). Opal dyes 620, 690, 570, and 520 were used to detect CD8, CD3, FoxP3, and CD4 correspondingly. Coverslips were mounted to slides with ProLong Diamond Antifade mounting media (#P36961, Thermo Fisher Scientific). Stained samples were scanned at 4X low magnification, and 10 high-powered fields were used at 20X magnification. High-powered fields were randomly selected within tumor areas identified on adjacent serial section hematoxylin and eosin stained slides by the pathologist. The spectral library was used to spectrally unmix images in the In Form software (2.4.10, Akoya Biosciences) for visualization of each color and to assess quality of each image. An algorithm was trained to unmix images, to segment cells, and identify and calculate CD3^+^, CD8^+^, and CD4^+^, and FoxP3^+^ cells. The data were then exported and analyzed in R where T cell population densities (number of positive cells/μm^2^) were determined. Similarly, to perform TLS quantification, antibodies to CD8a (clone 4SM15, eBioscience, #14-0808-82, Waltham, MA, USA), CD4 (clone EPR19514, Abcam, #ab183685, Waltham, MA, USA), and CD19 (clone EPR23174-145, Abcam, #ab245235) were used together with DAPI. Stained sections were scanned at 10x magnification, and manual TLS quantification was performed.

### Single-cell RNA sequencing

A total of 1.8 × 10^5^ to 6 × 10^5^ sorted CD45^+^ cells from each CRLM sample were diluted as necessary for appropriate input into the 10x Chromium chip (cell viability: 75 to 90%) (*n* = 3 mice per group) (10x Genomics, Pleasanton, CA, USA). The 10x Genomics Chromium Next GEM Single Cell 5′ v2 (Dual Index) workflow was used to generate TCR and 5′ Gene Expression (GEX) libraries. All processing and library construction steps were performed according to 10x Genomics User Guide CG000331 Rev C. Final amplified libraries were purified, quantified and average fragment sizes confirmed by gel electrophoresis using 4200 TapeStation and HS D5000 Screen Tape (Agilent). V(D)J and GEX libraries were pooled at the ratio 1:5 to achieve a respective sequencing depth of 5000 and 20,000 reads per cell. Paired-end, dual indexing sequencing was performed using a NovaSeq 6000 Illumina sequencing system, S4 flow cell. Raw reads were demultiplexed, and single-cell gene expression and V(D)J regions were quantified using 10x Genomics Cell Ranger 5.0.1 with the mm10 references as supplied by 10x Genomics and downloaded after 19 November 2020 (*73*). We used the SCANPY (*74*) data analysis pipeline for preprocessing and analysis of scRNA-seq data, with the following software versions: scanpy 1.4.6, anndata 0.7.1, umap 0.4.1, numpy 1.18.1, scipy 1.4.1, pandas 1.0.3, scikit-learn 0.21.2, statsmodels 0.10.1, and python-igraph 0.8.0. We applied the following cutoffs for filtering high-quality cells: <10% mitochondrial genes and >100 and <6000 genes expressed per cell, and excluded platelets, red blood cells, and doublets. We filtered out ribosomal genes and genes detected in less than three cells. Following sequencing alignment, preprocessing, quality control, and doublet removal, we recovered 20,287 cells from all samples combined, corresponding to greater than 1500 cells per sample. We log_2_ plus one transformed, normalized the data to 10,000 counts per cell, and scaled genes to unit variance. We performed batch correction using the highly variable genes (3469 genes) with ComBat (*75*) and principal components analysis with SCANPY (using the top 25 principal components). We then performed k-nearest neighbor graph construction and Leiden clustering on gene expression data. For analysis of all immune cells, we clustered cells with a resolution of 0.5. We reclustered on myeloid or T cells individually, removing any contaminating cells (nonmyeloid or non–T cell); for myeloid cells, we used a resolution of 0.3; for T cells, we used a resolution of 0.5. For differential expression between experimental groups, we used MAST to calculate fold change and significance, based on a model incorporating cellular detection rate (based on number of genes per cell) (*76*). For frequency proportions of cell types, a one-way analysis of variance (ANOVA) and Tukey’s multiple comparisons test was used for multiple comparisons across groups using Prism v9 software (GraphPad).

### TCR sequencing analysis

We further processed TCR sequencing data by selecting the most abundant TCR alpha or TCR beta clonotype for each cell barcode as previously described (*77*). We queried CDR3β motif sequences for AH1-specific CD8^+^ T cells. Nine of the sequences matching AH1-specific motifs were repeated 21 times: CASSEGQYEQYF; CASSLRLGGYAEQFF; CASSDGDYEQYF; CASSDGGYEQYF; CASSDAQYEQYF; CASSIKTGGFAEQFF; CASSSRTGGYAEQFF; CASSIKTGGYAEQFF; CASSPRDRNTEVFF (*78*–*82*). For the clonality analysis, only cells with a paired TCR alpha and beta chain and matched to a T cell in the single-cell data object were used for downstream analysis. The frequency of highly clonal T cells in each sample was compared using an unpaired *t* test in Prism v9 (GraphPad).

### Bulk RNA validation

We retrieved bulk transcriptome data from 396 primary colon cancer samples within the TCGA-COAD project (*20*) using the “TCGAbiolinks” package (*83*–*85*). Expression data were analyzed in R Studio (RStudio Inc., Boston, MA). We used the combined expression levels of *TNFSF14* (LIGHT) and *CTLA4* for patient stratification, which served as surrogates of *TNFSF14*-induced overexpression and CTLA-4 inhibition, respectively. Expression thresholds for high and low levels were determined using median values. The MCP-counter function (*86*) was applied to compute T cell exhaustion scores, using the following genes related to exhaustion: *HAVCR2*, *ENTPD1*, *LAG3*, *TIGIT*, *PDCD1*, *CTLA4*, and *TOX*. Similarly, a macrophage score was also assessed by evaluating expression levels of genes associated with the macrophage subsets seen in our mouse studies: *ARG1*, *CD68*, and *C1QA*. Statistical significance of observed differences was calculated using the Wilcoxon rank sum test, with a *P* value threshold of <0.05. Plots were generated using the ggplot2 and ggsignif packages.

### Mass cytometry (CyTOF)

CD45^+^ leukocytes were isolated with fluorescence-activated cell sorting as for scRNA-seq. The sorted CD45^+^ cells from three samples of each group were pooled together and stimulated with PMA at 20 ng/ml plus ionomycin at 1 μg/ml for 4 hours at 37°C in the presence of a transcription inhibitor (eBioscience, San Diego, CA, USA). We then followed the procedure for Maxpar Nuclear Antigen Staining as described by the manufacturer. The samples were run using a Helios mass cytometer (Fluidigm Inc., South San Francisco, CA, USA). All CyTOF files were normalized, and beads were removed from downstream analysis using the premessa R package (https://github.com/ParkerICI/premessa). Singlets were gated by event length and DNA using Cell Engine. Live cells were identified by cisplatin-negative cells. Manually gated T cells and manually gated CD8^+^ T cells were imported as a single-cell experiment (SCE) object using the CATALYST R package. Expression heatmaps were generated from the SCE objects. CATALYST’s cluster function was used to perform FlowSOM clustering (*k* = 10) on the T cell SCE. Manual cluster merging based on marker expression was used to consolidate the clusters in agreement with the delta plot (*k* = 8). Marker expression was used to identify clusters as certain T cell subsets. Plots of abundances of immune cell subsets were generated using manually gated data from Cell Engine (CellCarta) and the ggplot2 package in R.

### Real-time quantitative RT-PCR assay

RNA was extracted from surgically dissected CRLMs. One milliliter of TRIzol reagent (Invitrogen) per 50 to 100 mg of tissue was added to tumor tissue after snap-frozen specimens were pulverized (Biopulverizer, BioSpec Products). Chloroform (0.2 ml) per 1 ml of TRIzol reagent was added to dissociate nucleoprotein complexes. The RNA pellet was washed and dissolved in RNase-free water. Five micrograms of total RNA was reverse-transcribed into cDNA by using the High-Capacity cDNA Reverse Transcription Kit (AB Applied Biosystems, Waltham, MA, USA), and real-time quantitative PCR analysis was performed (QuantStudio 6 Real-Time PCR system). Each cDNA sample was amplified in triplicate for *Madcam1*, *Ccl21*, *Ccl4*, *Cxcl9*, *Cxcl10*, *Cxcl11*, *Ifng*, and *Gapdh* using the Fast SYBR Green Master Mixture Kit following the manufacturer’s instructions (AB Applied Biosystems). PCR primer sequences are summarized in table S3. PCR conditions were 120 s at 95°C, 15 s at 95°C, and 60 s at 60°C for 40 cycles. The concentration of the target gene was determined by the comparative CT method and normalized to the internal GAPDH (glyceraldehyde-3-phosphate dehydrogenase) control.

### Statistical analysis

Statistical analysis was performed by two-tailed Student’s *t* test for flow cytometry results, in vitro T cell proliferation, and T_reg_ suppression cell assays, except as noted in figure legends. All data are reported as means ± SEM. For scRNA-seq data, all *P* values were adjusted for false discovery rate (FDR) and significance was only shown for differential expression comparisons with 0.2 log-fold change or greater.

## References

[1] R. L. Siegel, K. D. Miller, H. E. Fuchs, A. Jemal, Cancer statistics, 2022. CA Cancer J. Clin. 72, 7–33 (2022). 35020204 10.3322/caac.21708

[2] J. S. Tomlinson, W. R. Jarnagin, R. P. DeMatteo, Y. Fong, P. Kornprat, M. Gonen, N. Kemeny, M. F. Brennan, L. H. Blumgart, M. D’Angelica, Actual 10-year survival after resection of colorectal liver metastases defines cure. J. Clin. Oncol. 25, 4575–4580 (2007). 17925551 10.1200/JCO.2007.11.0833

[3] A. V. Maker, G. Q. Phan, P. Attia, J. C. Yang, R. M. Sherry, S. L. Topalian, U. S. Kammula, R. E. Royal, L. R. Haworth, C. Levy, D. Kleiner, S. A. Mavroukakis, M. Yellin, S. A. Rosenberg, Tumor regression and autoimmunity in patients treated with cytotoxic T lymphocyte-associated antigen 4 blockade and interleukin 2: A phase I/II study. Ann. Surg. Oncol. 12, 1005–1016 (2005). 16283570 10.1245/ASO.2005.03.536 PMC1473970

[4] S. L. Topalian, F. S. Hodi, J. R. Brahmer, S. N. Gettinger, D. C. Smith, D. F. McDermott, J. D. Powderly, R. D. Carvajal, J. A. Sosman, M. B. Atkins, P. D. Leming, D. R. Spigel, S. J. Antonia, L. Horn, C. G. Drake, D. M. Pardoll, L. Chen, W. H. Sharfman, R. A. Anders, J. M. Taube, T. L. McMiller, H. Xu, A. J. Korman, M. Jure-Kunkel, S. Agrawal, D. McDonald, G. D. Kollia, A. Gupta, J. M. Wigginton, M. Sznol, Safety, activity, and immune correlates of anti-PD-1 antibody in cancer. N. Engl. J. Med. 366, 2443–2454 (2012). 22658127 10.1056/NEJMoa1200690 PMC3544539

[5] K. Y. Chung, I. Gore, L. Fong, A. Venook, S. B. Beck, P. Dorazio, P. J. Criscitiello, D. I. Healey, B. Huang, J. Gomez-Navarro, L. B. Saltz, Phase II study of the anti-cytotoxic T-lymphocyte-associated antigen 4 monoclonal antibody, tremelimumab, in patients with refractory metastatic colorectal cancer. J. Clin. Oncol. 28, 3485–3490 (2010). 20498386 10.1200/JCO.2010.28.3994

[6] D. T. Le, J. N. Uram, H. Wang, B. R. Bartlett, H. Kemberling, A. D. Eyring, A. D. Skora, B. S. Luber, N. S. Azad, D. Laheru, B. Biedrzycki, R. C. Donehower, A. Zaheer, G. A. Fisher, T. S. Crocenzi, J. J. Lee, S. M. Duffy, R. M. Goldberg, A. de la Chapelle, M. Koshiji, F. Bhaijee, T. Huebner, R. H. Hruban, L. D. Wood, N. Cuka, D. M. Pardoll, N. Papadopoulos, K. W. Kinzler, S. Zhou, T. C. Cornish, J. M. Taube, R. A. Anders, J. R. Eshleman, B. Vogelstein, L. A. Diaz Jr., PD-1 blockade in tumors with mismatch-repair deficiency. N. Engl. J. Med. 372, 2509–2520 (2015). 26028255 10.1056/NEJMoa1500596 PMC4481136

[7] M. R. Parkhurst, J. C. Yang, R. C. Langan, M. E. Dudley, D. A. Nathan, S. A. Feldman, J. L. Davis, R. A. Morgan, M. J. Merino, R. M. Sherry, M. S. Hughes, U. S. Kammula, G. Q. Phan, R. M. Lim, S. A. Wank, N. P. Restifo, P. F. Robbins, C. M. Laurencot, S. A. Rosenberg, T cells targeting carcinoembryonic antigen can mediate regression of metastatic colorectal cancer but induce severe transient colitis. Mol. Ther. 19, 620–626 (2011). 21157437 10.1038/mt.2010.272 PMC3048186

[8] F. C. Thistlethwaite, D. E. Gilham, R. D. Guest, D. G. Rothwell, M. Pillai, D. J. Burt, A. J. Byatte, N. Kirillova, J. W. Valle, S. K. Sharma, K. A. Chester, N. B. Westwood, S. E. R. Halford, S. Nabarro, S. Wan, E. Austin, R. E. Hawkins, The clinical efficacy of first-generation carcinoembryonic antigen (CEACAM5)-specific CAR T cells is limited by poor persistence and transient pre-conditioning-dependent respiratory toxicity. Cancer Immunol. Immunother. 66, 1425–1436 (2017). 28660319 10.1007/s00262-017-2034-7 PMC5645435

[9] I. H. Sahin, K. K. Ciombor, L. A. Diaz, J. Yu, R. Kim, Immunotherapy for microsatellite stable colorectal cancers: Challenges and novel therapeutic avenues. Am. Soc. Clin. Oncol. Educ. Book 42, 1–12 (2022). 10.1200/EDBK_349811 35658496

[10] P. Yu, Y. X. Fu, Targeting tumors with LIGHT to generate metastasis-clearing immunity. Cytokine Growth Factor Rev. 19, 285–294 (2008). 18508404 10.1016/j.cytogfr.2008.04.004 PMC2517180

[11] P. Yu, Y. Lee, W. Liu, R. K. Chin, J. Wang, Y. Wang, A. Schietinger, M. Philip, H. Schreiber, Y.-X. Fu, Priming of naive T cells inside tumors leads to eradication of established tumors. Nat. Immunol. 5, 141–149 (2004). 14704792 10.1038/ni1029

[12] W. Kang, Z. Feng, J. Luo, Z. He, J. Liu, J. Wu, P. Rong, Tertiary lymphoid structures in cancer: The double-edged sword role in antitumor immunity and potential therapeutic induction strategies. Front. Immunol. 12, 689270 (2021). 34394083 10.3389/fimmu.2021.689270 PMC8358404

[13] A. V. Maker, H. Ito, Q. Mo, E. Weisenberg, L.-X. Qin, S. Turcotte, S. Maithel, J. Shia, L. Blumgart, Y. Fong, W. R. Jarnagin, R. P. DeMatteo, M. I. D'Angelica, Genetic evidence that intratumoral T cell proliferation and activation are associated with recurrence and survival in patients with resected colorectal liver metastases. Cancer Immunol. Res. 3, 380–388 (2015). 25600439 10.1158/2326-6066.CIR-14-0212 PMC4390462

[14] G. Di Caro, F. Bergomas, F. Grizzi, A. Doni, P. Bianchi, A. Malesci, L. Laghi, P. Allavena, A. Mantovani, F. Marchesi, Occurrence of tertiary lymphoid tissue is associated with T cell infiltration and predicts better prognosis in early-stage colorectal cancers. Clin. Cancer Res. 20, 2147–2158 (2014). 24523438 10.1158/1078-0432.CCR-13-2590

[15] G. Qiao, J. Qin, N. Kunda, J. F. Calata, D. L. Mahmud, P. Gann, Y.-X. Fu, S. A. Rosenberg, B. S. Prabhakar, A. V. Maker, LIGHT elevation enhances immune eradication of colon cancer metastases. Cancer Res. 77, 1880–1891 (2017). 28249900 10.1158/0008-5472.CAN-16-1655 PMC5410174

[16] J. Z. Qin, V. Upadhyay, B. Prabhakar, A. V. Maker, Shedding LIGHT (TNFSF14) on the tumor microenvironment of colorectal cancer liver metastases. J. Transl. Med. 11, 70 (2013). 23514280 10.1186/1479-5876-11-70 PMC3623860

[17] J. Duraiswamy, K. M. Kaluza, G. J. Freeman, G. Coukos, Dual blockade of PD-1 and CTLA-4 combined with tumor vaccine effectively restores T cell rejection function in tumors. Cancer Res. 73, 3591–3603 (2013). 23633484 10.1158/0008-5472.CAN-12-4100 PMC3686913

[18] J. Qin, N. M. Kunda, G. Qiao, K. Tulla, B. S. Prabhakar, A. V. Maker, Vaccination with mitoxantrone-treated primary colon cancer cells enhances tumor-infiltrating lymphocytes and clinical responses in colorectal liver metastases. J. Surg. Res. 233, 57–64 (2019). 30502288 10.1016/j.jss.2018.07.068

[19] J. C. Castle, M. Loewer, S. Boegel, J. de Graaf, C. Bender, A. D. Tadmor, V. Boisguerin, T. Bukur, P. Sorn, C. Paret, M. Diken, S. Kreiter, Ö. Türeci, U. Sahin, Immunomic, genomic and transcriptomic characterization of CT26 colorectal carcinoma. BMC Genomics 15, 190 (2014). 24621249 10.1186/1471-2164-15-190 PMC4007559

[20] The Cancer Genome Atlas Network, Comprehensive molecular characterization of human colon and rectal cancer. Nature 487, 330–337 (2012). 22810696 10.1038/nature11252 PMC3401966

[21] W. W. Ho, I. L. Gomes-Santos, S. Aoki, M. Datta, K. Kawaguchi, N. P. Talele, S. Roberge, J. Ren, H. Liu, I. X. Chen, P. Andersson, S. Chatterjee, A. S. Kumar, Z. Amoozgar, Q. Zhang, P. Huang, M. R. Ng, V. P. Chauhan, L. Xu, D. G. Duda, J. W. Clark, M. J. Pittet, D. Fukumura, R. K. Jain, Dendritic cell paucity in mismatch repair-proficient colorectal cancer liver metastases limits immune checkpoint blockade efficacy. Proc. Natl. Acad. Sci. U.S.A. 118, e2105323118 (2021). 34725151 10.1073/pnas.2105323118 PMC8609309

[22] M. Ramachandran, A. Vaccaro, T. van de Walle, M. Georganaki, R. Lugano, K. Vemuri, D. Kourougkiaouri, K. Vazaios, M. Hedlund, G. Tsaridou, L. Uhrbom, I. Pietilä, M. Martikainen, L. van Hooren, T. Olsson Bontell, A. S. Jakola, D. Yu, B. Westermark, M. Essand, A. Dimberg, Tailoring vascular phenotype through AAV therapy promotes anti-tumor immunity in glioma. Cancer Cell 41, 1134–1151.e10 (2023). 37172581 10.1016/j.ccell.2023.04.010

[23] D. Coppola, M. Nebozhyn, F. Khalil, H. Dai, T. Yeatman, A. Loboda, J. J. Mulé, Unique ectopic lymph node-like structures present in human primary colorectal carcinoma are identified by immune gene array profiling. Am. J. Pathol. 179, 37–45 (2011). 21703392 10.1016/j.ajpath.2011.03.007 PMC3123872

[24] J.-P. Girard, C. Moussion, R. Förster, HEVs, lymphatics and homeostatic immune cell trafficking in lymph nodes. Nat. Rev. Immunol. 12, 762–773 (2012). 23018291 10.1038/nri3298

[25] I. G. House, P. Savas, J. Lai, A. X. Y. Chen, A. J. Oliver, Z. L. Teo, K. L. Todd, M. A. Henderson, L. Giuffrida, E. V. Petley, K. Sek, S. Mardiana, T. N. Gide, C. Quek, R. A. Scolyer, G. V. Long, J. S. Wilmott, S. Loi, P. K. Darcy, P. A. Beavis, Macrophage-derived CXCL9 and CXCL10 are required for antitumor immune responses following immune checkpoint blockade. Clin. Cancer Res. 26, 487–504 (2020). 31636098 10.1158/1078-0432.CCR-19-1868

[26] K. Van Raemdonck, P. E. Van den Steen, S. Liekens, J. Van Damme, S. Struyf, CXCR3 ligands in disease and therapy. Cytokine Growth Factor Rev. 26, 311–327 (2015). 25498524 10.1016/j.cytogfr.2014.11.009

[27] R. J. Lim, R. Salehi-Rad, L. M. Tran, M. S. Oh, C. Dumitras, W. P. Crosson, R. Li, T. S. Patel, S. Man, C. E. Yean, J. Abascal, Z. Huang, S. L. Ong, K. Krysan, S. M. Dubinett, B. Liu, CXCL9/10-engineered dendritic cells promote T cell activation and enhance immune checkpoint blockade for lung cancer. Cell Rep. Med. 5, 101479 (2024). 38518770 10.1016/j.xcrm.2024.101479 PMC11031384

[28] P. V. Kharchenko, L. Silberstein, D. T. Scadden, Bayesian approach to single-cell differential expression analysis. Nat. Methods 11, 740–742 (2014). 24836921 10.1038/nmeth.2967 PMC4112276

[29] P. Pathria, T. L. Louis, J. A. Varner, Targeting tumor-associated macrophages in cancer. Trends Immunol. 40, 310–327 (2019). 30890304 10.1016/j.it.2019.02.003

[30] J. Zhou, Z. Tang, S. Gao, C. Li, Y. Feng, X. Zhou, Tumor-associated macrophages: Recent insights and therapies. Front. Oncol. 10, 188 (2020). 32161718 10.3389/fonc.2020.00188 PMC7052362

[31] L. T. Roumenina, M. V. Daugan, R. Noé, F. Petitprez, Y. A. Vano, R. Sanchez-Salas, E. Becht, J. Meilleroux, B. L. Clec'h, N. A. Giraldo, N. S. Merle, C.-M. Sun, V. Verkarre, P. Validire, J. Selves, L. Lacroix, O. Delfour, I. Vandenberghe, C. Thuilliez, S. Keddani, I. B. Sakhi, E. Barret, P. Ferré, N. Corvaïa, A. Passioukov, E. Chetaille, M. Botto, A. de Reynies, S. M. Oudard, A. Mejean, X. Cathelineau, C. Sautès-Fridman, W. H. Fridman, Tumor cells hijack macrophage-produced complement C1q to promote tumor growth. Cancer Immunol. Res. 7, 1091–1105 (2019). 31164356 10.1158/2326-6066.CIR-18-0891

[32] M. Binnewies, J. L. Pollack, J. Rudolph, S. Dash, M. Abushawish, T. Lee, N. S. Jahchan, P. Canaday, E. Lu, M. Norng, S. Mankikar, V. M. Liu, X. Du, A. Chen, R. Mehta, R. Palmer, V. Juric, L. Liang, K. P. Baker, L. Reyno, M. F. Krummel, M. Streuli, V. Sriram, Targeting TREM2 on tumor-associated macrophages enhances immunotherapy. Cell Rep. 37, 109844 (2021). 34686340 10.1016/j.celrep.2021.109844

[33] J. Wang, N. Zhu, X. Su, Y. Gao, R. Yang, Novel tumor-associated macrophage populations and subpopulations by single cell RNA sequencing. Front. Immunol. 14, 1264774 (2024). 38347955 10.3389/fimmu.2023.1264774 PMC10859433

[34] I. Vitale, G. Manic, L. M. Coussens, G. Kroemer, L. Galluzzi, Macrophages and metabolism in the tumor microenvironment. Cell Metab. 30, 36–50 (2019). 31269428 10.1016/j.cmet.2019.06.001

[35] S. A. Rosenberg, Interleukin-2 and the development of immunotherapy for the treatment of patients with cancer. Cancer J. Sci. Am. 6, S2–S7 (2000). 10685650

[36] Z. Fan, P. Yu, Y. Wang, Y. Wang, M. L. Fu, W. Liu, Y. Sun, Y. X. Fu, NK-cell activation by LIGHT triggers tumor-specific CD8^+^ T cell immunity to reject established tumors. Blood 107, 1342–1351 (2006). 16223768 10.1182/blood-2005-08-3485 PMC1895398

[37] A. V. Maker, Precise identification of immunotherapeutic targets for solid malignancies using clues within the tumor microenvironment-Evidence to turn on the LIGHT. Oncoimmunology 5, e1069937 (2016). 26942091 10.1080/2162402X.2015.1069937 PMC4760328

[38] J. Yu, M. D. Green, S. Li, Y. Sun, S. N. Journey, J. E. Choi, S. M. Rizvi, A. Qin, J. J. Waninger, X. Lang, Z. Chopra, I. El Naqa, J. Zhou, Y. Bian, L. Jiang, A. Tezel, J. Skvarce, R. K. Achar, M. Sitto, B. S. Rosen, F. Su, S. P. Narayanan, X. Cao, S. Wei, W. Szeliga, L. Vatan, C. Mayo, M. A. Morgan, C. A. Schonewolf, K. Cuneo, I. Kryczek, V. T. Ma, C. D. Lao, T. S. Lawrence, N. Ramnath, F. Wen, A. M. Chinnaiyan, M. Cieslik, A. Alva, W. Zou, Liver metastasis restrains immunotherapy efficacy via macrophage-mediated T cell elimination. Nat. Med. 27, 152–164 (2021). 33398162 10.1038/s41591-020-1131-x PMC8095049

[39] S. C. Wei, C. R. Duffy, J. P. Allison, Fundamental mechanisms of immune checkpoint blockade therapy. Cancer Discov. 8, 1069–1086 (2018). 30115704 10.1158/2159-8290.CD-18-0367

[40] X. Cao, S. F. Cai, T. A. Fehniger, J. Song, L. I. Collins, D. R. Piwnica-Worms, T. J. Ley, Granzyme B and perforin are important for regulatory T cell-mediated suppression of tumor clearance. Immunity 27, 635–646 (2007). 17919943 10.1016/j.immuni.2007.08.014

[41] D. I. Gabrilovich, S. Nagaraj, Myeloid-derived suppressor cells as regulators of the immune system. Nat. Rev. Immunol. 9, 162–174 (2009). 19197294 10.1038/nri2506 PMC2828349

[42] L. N. Shi, Y. Zhou, C. Wu, W. Huang, F. Yuan, J. Chen, Z. Wu, W. Tu, H. Chen, Q. Chen, M. Zhu, H. Peng, Y. Yang, H. Tang, LIGHT of pulmonary NKT cells annihilates tissue protective alveolar macrophages in augmenting severe influenza pneumonia. Sci. Bull. (Beijing) 66, 2124–2134 (2021). 36654270 10.1016/j.scib.2021.01.026

[43] M. L. Petreaca, M. Yao, C. Ware, M. M. Martins-Green, Vascular endothelial growth factor promotes macrophage apoptosis through stimulation of tumor necrosis factor superfamily member 14 (TNFSF14/LIGHT). Wound Repair Regen. 16, 602–614 (2008). 19128255 10.1111/j.1524-475X.2008.00411.x

[44] J. S. Weber, M. S. Carlino, A. Khattak, T. Meniawy, G. Ansstas, M. H. Taylor, K. B. Kim, M. McKean, G. V. Long, R. J. Sullivan, M. Faries, T. T. Tran, C. L. Cowey, A. Pecora, M. Shaheen, J. Segar, T. Medina, V. Atkinson, G. T. Gibney, J. J. Luke, S. Thomas, E. I. Buchbinder, J. A. Healy, M. Huang, M. Morrissey, I. Feldman, V. Sehgal, C. Robert-Tissot, P. Hou, L. Zhu, M. Brown, P. Aanur, R. S. Meehan, T. Zaks, Individualised neoantigen therapy mRNA-4157 (V940) plus pembrolizumab versus pembrolizumab monotherapy in resected melanoma (KEYNOTE-942): A randomised, phase 2b study. Lancet 403, 632–644 (2024). 38246194 10.1016/S0140-6736(23)02268-7

[45] L. A. Rojas, Z. Sethna, K. C. Soares, C. Olcese, N. Pang, E. Patterson, J. Lihm, N. Ceglia, P. Guasp, A. Chu, R. Yu, A. K. Chandra, T. Waters, J. Ruan, M. Amisaki, A. Zebboudj, Z. Odgerel, G. Payne, E. Derhovanessian, F. Müller, I. Rhee, M. Yadav, A. Dobrin, M. Sadelain, M. Łuksza, N. Cohen, L. Tang, O. Basturk, M. Gönen, S. Katz, R. K. Do, A. S. Epstein, P. Momtaz, W. Park, R. Sugarman, A. M. Varghese, E. Won, A. Desai, A. C. Wei, M. I. D'Angelica, T. P. Kingham, I. Mellman, T. Merghoub, J. D. Wolchok, U. Sahin, O. Türeci, B. D. Greenbaum, W. R. Jarnagin, J. Drebin, E. M. O'Reilly, V. P. Balachandran, Personalized RNA neoantigen vaccines stimulate T cells in pancreatic cancer. Nature 618, 144–150 (2023). 37165196 10.1038/s41586-023-06063-y PMC10171177

[46] R. E. McIntyre, S. J. A. Buczacki, M. J. Arends, D. J. Adams, Mouse models of colorectal cancer as preclinical models. Bioessays 37, 909–920 (2015). 26115037 10.1002/bies.201500032 PMC4755199

[47] V. K. Mittal, J. S. Bhullar, K. Jayant, Animal models of human colorectal cancer: Current status, uses and limitations. World J. Gastroenterol. 21, 11854–11861 (2015). 26557009 10.3748/wjg.v21.i41.11854 PMC4631983

[48] J. P. Evans, B. K. Winiarski, P. A. Sutton, L. Ressel, C. A. Duckworth, D. M. Pritchard, D. H. Palmer, C. E. Goldring, N. R. Kitteringham, Development of an orthotopic syngeneic murine model of colorectal cancer for use in translational research. Lab. Anim 53, 598–609 (2019). 30760081 10.1177/0023677219826165 PMC6900214

[49] F. Bürtin, C. S. Mullins, M. Linnebacher, Mouse models of colorectal cancer: Past, present and future perspectives. World J. Gastroenterol. 26, 1394–1426 (2020). 32308343 10.3748/wjg.v26.i13.1394 PMC7152519

[50] J. C. Lee, S. Mehdizadeh, J. Smith, A. Young, I. A. Mufazalov, C. T. Mowery, A. Daud, J. A. Bluestone, Regulatory T cell control of systemic immunity and immunotherapy response in liver metastasis. Sci. Immunol. 5, eaba0759 (2020). 33008914 10.1126/sciimmunol.aba0759 PMC7755924

[51] T. Huu Hoang, M. Sato-Matsubara, H. Yuasa, T. Matsubara, L. T. T. Thuy, H. Ikenaga, D. M. Phuong, N. V. Hanh, V. N. Hieu, D. V. Hoang, H. Hai, Y. Okina, M. Enomoto, A. Tamori, A. Daikoku, H. Urushima, K. Ikeda, N. Q. Dat, Y. Yasui, H. Shinkawa, S. Kubo, R. Yamagishi, N. Ohtani, K. Yoshizato, J. Gracia-Sancho, N. Kawada, Cancer cells produce liver metastasis via gap formation in sinusoidal endothelial cells through proinflammatory paracrine mechanisms. Sci. Adv. 8, eabo5525 (2022). 36170363 10.1126/sciadv.abo5525 PMC9519040

[52] L. B. Alexandrov, S. Nik-Zainal, D. C. Wedge, S. A. J. R. Aparicio, S. Behjati, A. V. Biankin, G. R. Bignell, N. Bolli, A. Borg, A.-L. Børresen-Dale, S. Boyault, B. Burkhardt, A. P. Butler, C. Caldas, H. R. Davies, C. Desmedt, R. Eils, J. E. Eyfjörd, J. A. Foekens, M. Greaves, F. Hosoda, B. Hutter, T. Ilicic, S. Imbeaud, M. Imielinski, N. Jäger, D. T. W. Jones, D. Jones, S. Knappskog, M. Kool, S. R. Lakhani, C. López-Otín, S. Martin, N. C. Munshi, H. Nakamura, P. A. Northcott, M. Pajic, E. Papaemmanuil, A. Paradiso, J. V. Pearson, X. S. Puente, K. Raine, M. Ramakrishna, A. L. Richardson, J. Richter, P. Rosenstiel, M. Schlesner, T. N. Schumacher, P. N. Span, J. W. Teague, Y. Totoki, A. N. J. Tutt, R. Valdés-Mas, M. M. van Buuren, L. van ‘t Veer, A. Vincent-Salomon, N. Waddell, L. R. Yates, Australian Pancreatic Cancer Genome Initiative, ICGC Breast Cancer Consortium, ICGC MMML-Seq Consortium, ICGC Ped Brain, J. Zucman-Rossi, P. A. Futreal, U. M. Dermott, P. Lichter, M. Meyerson, S. M. Grimmond, R. Siebert, E. Campo, T. Shibata, S. M. Pfister, P. J. Campbell, M. R. Stratton, Signatures of mutational processes in human cancer. Nature 500, 415–421 (2013). 23945592 10.1038/nature12477 PMC3776390

[53] D. Mouradov, C. Sloggett, R. N. Jorissen, C. G. Love, S. Li, A. W. Burgess, D. Arango, R. L. Strausberg, D. Buchanan, S. Wormald, L. O'Connor, J. L. Wilding, D. Bicknell, I. P. M. Tomlinson, W. F. Bodmer, J. M. Mariadason, O. M. Sieber, Colorectal cancer cell lines are representative models of the main molecular subtypes of primary cancer. Cancer Res. 74, 3238–3247 (2014). 24755471 10.1158/0008-5472.CAN-14-0013

[54] M. Efremova, D. Rieder, V. Klepsch, P. Charoentong, F. Finotello, H. Hackl, N. Hermann-Kleiter, M. Löwer, G. Baier, A. Krogsdam, Z. Trajanoski, Targeting immune checkpoints potentiates immunoediting and changes the dynamics of tumor evolution. Nat. Commun. 9, 32 (2018). 29296022 10.1038/s41467-017-02424-0 PMC5750210

[55] S. F. Cai, T. A. Fehniger, X. Cao, J. C. Mayer, J. D. Brune, A. R. French, T. J. Ley, Differential expression of granzyme B and C in murine cytotoxic lymphocytes. J. Immunol. 182, 6287–6297 (2009). 19414782 10.4049/jimmunol.0804333 PMC2714542

[56] T. A. Fehniger, S. F. Cai, X. Cao, A. J. Bredemeyer, R. M. Presti, A. R. French, T. J. Ley, Acquisition of murine NK cell cytotoxicity requires the translation of a pre-existing pool of granzyme B and perforin mRNAs. Immunity 26, 798–811 (2007). 17540585 10.1016/j.immuni.2007.04.010

[57] J. Martin, R. H. Jenkins, R. Bennagi, A. Krupa, A. O. Phillips, T. Bowen, D. J. Fraser, Post-transcriptional regulation of Transforming Growth Factor Beta-1 by microRNA-744. PLOS ONE 6, e25044 (2011). 21991303 10.1371/journal.pone.0025044 PMC3186795

[58] F. F. Duan, G. Barron, A. Meliton, G. M. Mutlu, N. O. Dulin, L. Schuger, P311 promotes lung fibrosis via stimulation of Transforming Growth Factor-β1, -β2, and -β3 translation. Am. J. Respir. Cell Mol. Biol. 60, 221–231 (2019). 30230348 10.1165/rcmb.2018-0028OC PMC6376409

[59] Z. Deng, T. Fan, C. Xiao, H. Tian, Y. Zheng, C. Li, J. He, TGF-β signaling in health, disease and therapeutics. Signal Transduct. Target. Ther. 9, 61 (2024). 38514615 10.1038/s41392-024-01764-w PMC10958066

[60] K. O'Leary, Liver metastases cultivate an immune desert. Nat. Rev. Cancer 21, 143 (2021). 33547456 10.1038/s41568-021-00338-0

[61] G. Hu, Y. Liu, H. Li, D. Zhao, L. Yang, J. Shen, X. Hong, X. Cao, Q. Wang, Adenovirus-mediated LIGHT gene modification in murine B-cell lymphoma elicits a potent antitumor effect. Cell. Mol. Immunol. 7, 296–305 (2010). 20418899 10.1038/cmi.2010.15 PMC4003227

[62] W. Zou, H. Zheng, T. C. He, J. Chang, Y. X. Fu, W. Fan, LIGHT delivery to tumors by mesenchymal stem cells mobilizes an effective antitumor immune response. Cancer Res. 72, 2980–2989 (2012). 22511579 10.1158/0008-5472.CAN-11-4216

[63] S. Zhou, Y. Huang, Y. Chen, Y. Liu, L. Xie, Y. You, S. Tong, J. Xu, G. Jiang, Q. Song, N. Mei, F. Ma, X. Gao, H. Chen, J. Chen, Reprogramming systemic and local immune function to empower immunotherapy against glioblastoma. Nat. Commun. 14, 435 (2023). 36702831 10.1038/s41467-023-35957-8 PMC9880004

[64] H. Tang, Y. Wang, L. K. Chlewicki, Y. Zhang, J. Guo, W. Liang, J. Wang, X. Wang, Y. X. Fu, Facilitating T cell infiltration in tumor microenvironment overcomes resistance to PD-L1 blockade. Cancer Cell 30, 500 (2016). 27622338 10.1016/j.ccell.2016.08.011

[65] S. Dai, Y. Lv, W. Xu, Y. Yang, C. Liu, X. Dong, H. Zhang, B. S. Prabhakar, A. V. Maker, P. Seth, H. Wang, Oncolytic adenovirus encoding LIGHT (TNFSF14) inhibits tumor growth via activating anti-tumor immune responses in 4T1 mouse mammary tumor model in immune competent syngeneic mice. Cancer Gene Ther. 27, 923–933 (2020). 32307442 10.1038/s41417-020-0173-z

[66] B. Koscso, Z. Ao, C. Passaro, N. Shah, N. Ly, P. Timpug, B. A. Aksoy, D. Sun, D. J. Li, K.-L. Sheahan, V. Young, T. Ross, B. Primack, M. Langley, J. Tchaicha, D. K. Sethi, J. ter Meulen, M. Ols, Abstract LB065: Tumor-infiltrating lymphocytes (TIL) engineered with regulatable membrane-bound IL15 (mbIL15) and LIGHT (TNFSF14) show enhanced efficacy in fibroblast-containing cold tumors. Cancer Res. 84, LB065 (2024).

[67] F. S. Hodi, S. J. O'Day, D. F. McDermott, R. W. Weber, J. A. Sosman, J. B. Haanen, R. Gonzalez, C. Robert, D. Schadendorf, J. C. Hassel, W. Akerley, A. J. van den Eertwegh, J. Lutzky, P. Lorigan, J. M. Vaubel, G. P. Linette, D. Hogg, C. H. Ottensmeier, C. Lebbe, C. Peschel, I. Quirt, J. I. Clark, J. D. Wolchok, J. S. Weber, J. Tian, M. J. Yellin, G. M. Nichol, A. Hoos, W. J. Urba, Improved survival with ipilimumab in patients with metastatic melanoma. N. Engl. J. Med. 363, 711–723 (2010). 20525992 10.1056/NEJMoa1003466 PMC3549297

[68] G. K. Abou-Alfa, G. Lau, M. Kudo, S. L. Chan, R. K. Kelley, J. Furuse, W. Sukeepaisarnjaroen, Y.-K. Kang, T. V. Dao, E. N. De Toni, L. Rimassa, V. Breder, A. Vasilyev, A. Heurgué, V. C. Tam, K. Mody, S. C. Thungappa, Y. Ostapenko, T. Yau, S. Azevedo, M. Varela, A.-L. Cheng, S. Qin, P. R. Galle, S. Ali, M. Marcovitz, M. Makowsky, P. He, J. F. Kurland, A. Negro, B. Sangro, Tremelimumab plus Durvalumab in unresectable hepatocellular carcinoma. NEJM Evid. 1, EVIDoa2100070 (2022). 38319892 10.1056/EVIDoa2100070

[69] R. J. Motzer, N. M. Tannir, D. F. McDermott, O. Aren Frontera, B. Melichar, T. K. Choueiri, E. R. Plimack, P. Barthélémy, C. Porta, S. George, T. Powles, F. Donskov, V. Neiman, C. K. Kollmannsberger, P. Salman, H. Gurney, R. Hawkins, A. Ravaud, M.-O. Grimm, S. Bracarda, C. H. Barrios, Y. Tomita, D. Castellano, B. I. Rini, A. C. Chen, S. Mekan, M. B. McHenry, M. Wind-Rotolo, J. Doan, P. Sharma, H. J. Hammers, B. Escudier, CheckMate 214 Investigators, Nivolumab plus Ipilimumab versus Sunitinib in advanced renal-cell carcinoma. N. Engl. J. Med. 378, 1277–1290 (2018). 29562145 10.1056/NEJMoa1712126 PMC5972549

[70] T. André, E. Elez, H.-J. Lenz, L. H. Jensen, Y. Touchefeu, E. Van Cutsem, R. Garcia-Carbonero, D. Tougeron, G. A. Mendez, M. Schenker, C. de la Fouchardiere, M. L. Limon, T. Yoshino, J. Li, J. L. Manzano Mozo, L. Dahan, G. Tortora, M. Chalabi, E. Goekkurt, M. I. Braghiroli, R. Joshi, T. Cil, F. Aubin, E. Cela, T. Chen, M. Lei, L. Jin, S. I. Blum, S. Lonardi, Nivolumab plus ipilimumab versus nivolumab in microsatellite instability-high metastatic colorectal cancer (CheckMate 8HW): A randomised, open-label, phase 3 trial. Lancet 405, 383–395 (2025). 39874977 10.1016/S0140-6736(24)02848-4

[71] Z.-B. Hu, C.-T. Wu, H. Wang, Q.-W. Zhang, L. Wang, R.-L. Wang, Z.-Z. Lu, L.-S. Wang, A simplified system for generating oncolytic adenovirus vector carrying one or two transgenes. Cancer Gene Ther. 15, 173–182 (2008). 18157145 10.1038/sj.cgt.7701105

[72] T. Nasukawa, J. Uchiyama, S. Taharaguchi, S. Ota, T. Ujihara, S. Matsuzaki, H. Murakami, K. Mizukami, M. Sakaguchi, Virus purification by CsCl density gradient using general centrifugation. Arch. Virol. 162, 3523–3528 (2017). 28785814 10.1007/s00705-017-3513-z

[73] G. X. Y. Zheng, J. M. Terry, P. Belgrader, P. Ryvkin, Z. W. Bent, R. Wilson, S. B. Ziraldo, T. D. Wheeler, G. P. McDermott, J. Zhu, M. T. Gregory, J. Shuga, L. Montesclaros, J. G. Underwood, D. A. Masquelier, S. Y. Nishimura, M. Schnall-Levin, P. W. Wyatt, C. M. Hindson, R. Bharadwaj, A. Wong, K. D. Ness, L. W. Beppu, H. J. Deeg, C. McFarland, K. R. Loeb, W. J. Valente, N. G. Ericson, E. A. Stevens, J. P. Radich, T. S. Mikkelsen, B. J. Hindson, J. H. Bielas, Massively parallel digital transcriptional profiling of single cells. Nat. Commun. 8, 14049 (2017). 28091601 10.1038/ncomms14049 PMC5241818

[74] F. A. Wolf, P. Angerer, F. J. Theis, SCANPY: Large-scale single-cell gene expression data analysis. Genome Biol. 19, 15 (2018). 29409532 10.1186/s13059-017-1382-0 PMC5802054

[75] W. E. Johnson, C. Li, A. Rabinovic, Adjusting batch effects in microarray expression data using empirical Bayes methods. Biostatistics 8, 118–127 (2007). 16632515 10.1093/biostatistics/kxj037

[76] G. Finak, A. McDavid, M. Yajima, J. Deng, V. Gersuk, A. K. Shalek, C. K. Slichter, H. W. Miller, M. J. McElrath, M. Prlic, P. S. Linsley, R. Gottardo, MAST: A flexible statistical framework for assessing transcriptional changes and characterizing heterogeneity in single-cell RNA sequencing data. Genome Biol. 16, 278 (2015). 26653891 10.1186/s13059-015-0844-5 PMC4676162

[77] D. Y. Oh, S. S. Kwek, S. S. Raju, T. Li, E. McCarthy, E. Chow, D. Aran, A. Ilano, C.-C. S. Pai, C. Rancan, K. Allaire, A. Burra, Y. Sun, M. H. Spitzer, S. Mangul, S. Porten, M. V. Meng, T. W. Friedlander, C. J. Ye, L. Fong, Intratumoral CD4^+^ T cells mediate anti-tumor cytotoxicity in human bladder cancer. Cell 181, 1612–1625.e13 (2020). 32497499 10.1016/j.cell.2020.05.017 PMC7321885

[78] M. Stringhini, P. Probst, D. Neri, Immunotherapy of CT26 murine tumors is characterized by an oligoclonal response of tissue-resident memory T cells against the AH1 rejection antigen. Eur. J. Immunol. 50, 1591–1597 (2020). 32470143 10.1002/eji.201948433

[79] J. D. Buhrman, K. R. Jordan, D. J. Munson, B. L. Moore, J. W. Kappler, J. E. Slansky, Improving antigenic peptide vaccines for cancer immunotherapy using a dominant tumor-specific T cell receptor. J. Biol. Chem. 288, 33213–33225 (2013). 24106273 10.1074/jbc.M113.509554 PMC3829168

[80] P. Wei, K. R. Jordan, J. D. Buhrman, J. Lei, H. Deng, P. Marrack, S. Dai, J. W. Kappler, J. E. Slansky, L. Yin, Structures suggest an approach for converting weak self-peptide tumor antigens into superagonists for CD8 T cells in cancer. Proc. Natl. Acad. Sci. U.S.A. 118, e2100588118 (2021). 34074778 10.1073/pnas.2100588118 PMC8201969

[81] N. P. Rudqvist, K. A. Pilones, C. Lhuillier, E. Wennerberg, J. W. Sidhom, R. O. Emerson, H. S. Robins, J. Schneck, S. C. Formenti, S. Demaria, Radiotherapy and CTLA-4 blockade shape the TCR repertoire of tumor-infiltrating T cells. Cancer Immunol. Res. 6, 139–150 (2018). 29180535 10.1158/2326-6066.CIR-17-0134 PMC6020019

[82] K. R. Jordan, J. D. Buhrman, J. Sprague, B. L. Moore, D. Gao, J. W. Kappler, J. E. Slansky, TCR hypervariable regions expressed by T cells that respond to effective tumor vaccines. Cancer Immunol. Immunother. 61, 1627–1638 (2012). 22350070 10.1007/s00262-012-1217-5 PMC3410973

[83] M. Mounir, M. Lucchetta, T. C. Silva, C. Olsen, G. Bontempi, X. Chen, H. Noushmehr, A. Colaprico, E. Papaleo, New functionalities in the TCGAbiolinks package for the study and integration of cancer data from GDC and GTEx. PLOS Comput. Biol. 15, e1006701 (2019). 30835723 10.1371/journal.pcbi.1006701 PMC6420023

[84] A. Colaprico, T. C. Silva, C. Olsen, L. Garofano, C. Cava, D. Garolini, T. S. Sabedot, T. M. Malta, S. M. Pagnotta, I. Castiglioni, M. Ceccarelli, G. Bontempi, H. Noushmehr, TCGAbiolinks: An R/Bioconductor package for integrative analysis of TCGA data. Nucleic Acids Res. 44, e71 (2016). 26704973 10.1093/nar/gkv1507 PMC4856967

[85] T. C. Silva, A. Colaprico, C. Olsen, F. D'Angelo, G. Bontempi, M. Ceccarelli, H. Noushmehr, TCGA Workflow: Analyze cancer genomics and epigenomics data using Bioconductor packages. F1000Res 5, 1542 (2016). 28232861 10.12688/f1000research.8923.2 PMC5302158

[86] E. Becht, N. A. Giraldo, L. Lacroix, B. Buttard, N. Elarouci, F. Petitprez, J. Selves, P. Laurent-Puig, C. Sautès-Fridman, W. H. Fridman, A. de Reyniès, Estimating the population abundance of tissue-infiltrating immune and stromal cell populations using gene expression. Genome Biol. 17, 218 (2016). 27765066 10.1186/s13059-016-1070-5 PMC5073889

