# Supplementary material for: Combination LIGHT overexpression and checkpoint blockade disrupts the tumor immune environment impacting colorectal liver metastases
Source: Sci Adv. 2025 Oct 8;11(41):eadv9161. doi: 10.1126/sciadv.adv9161 (PMC12506966; doi:10.1126/sciadv.adv9161)
Supplement: Supplementary file 1 — Figs. S1 to S7 Tables S1 to S3 [file sciadv.adv9161_sm.pdf]

Science Advances
AAAS

Supplementary Materials for

Combination LIGHT overexpression and checkpoint blockade disrupts the tumor immune environment impacting colorectal liver metastases

Bridget P. Keenan *et al.*

Corresponding author: Ajay V. Maker, ajay.maker@ucsf.edu

Sci. Adv. **11**, eadv9161 (2025)
DOI: 10.1126/sciadv.adv9161

This PDF file includes:

Figs. S1 to S7
Tables S1 to S3

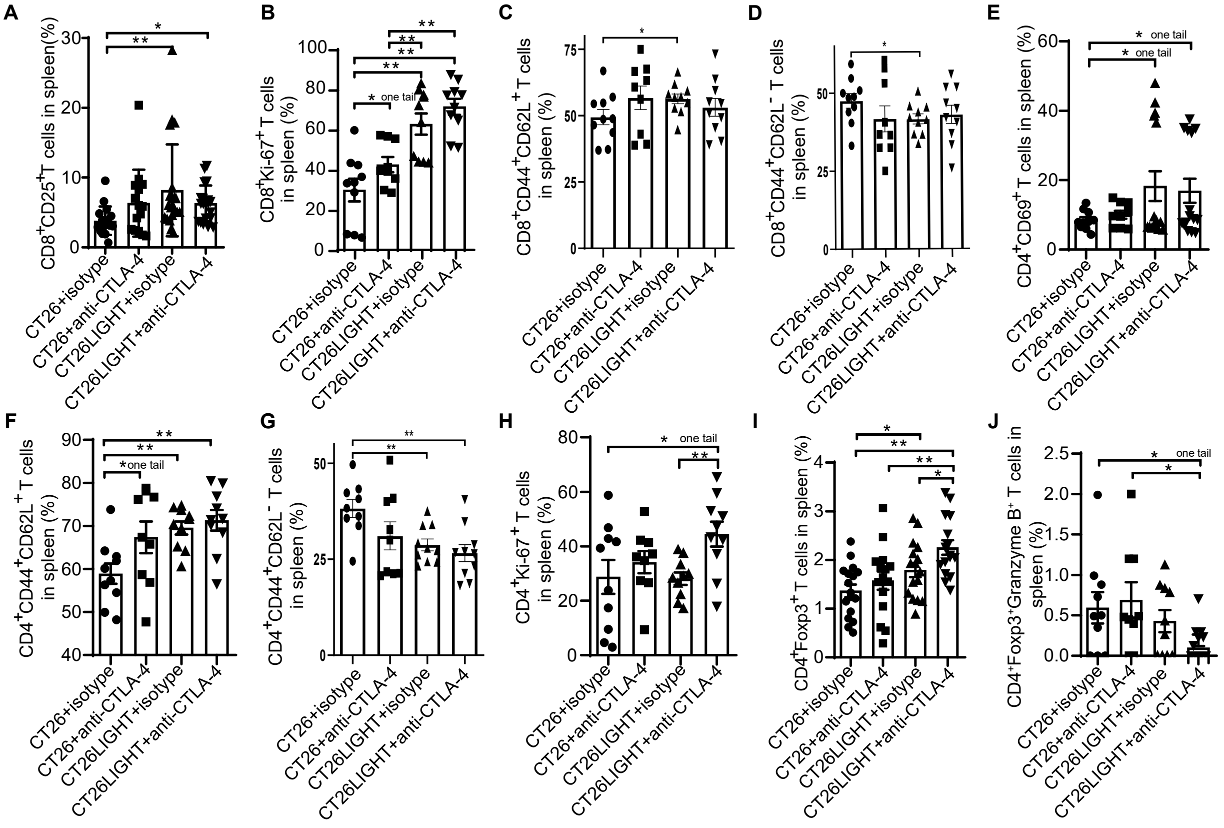

Figure S1. Combination treatment induces splenic $CD8^+$ and $CD4^+$ T cell activation and proliferation, and inhibits splenic Treg cell function. **(A)** Percentage of splenic $CD8^+CD25^+$ cells in mice from different treatment groups analyzed with flow cytometry analysis. **(B)** Percentage of splenic $CD8^+$Ki-67^+ in different treatment groups analyzed with flow cytometry analysis. **(C-D)** Percentage of splenic central memory $CD8^+$ T cells ($CD44^+CD62L^+$) and effector memory $CD8^+$ T cells ($CD44^+CD62L^-$) in different treatment groups analyzed with flow cytometry analysis. **(E)** Percentage of splenic $CD4^+CD69^+$ cells in different treatment groups analyzed with flow cytometry analysis. **(F-G)** Percentage of splenic central memory $CD4^+$ T cells ($CD44^+CD62L^+$) and effector memory $CD4^+$ T cells ($CD44^+CD62L^-$) in different treatment groups analyzed with flow cytometry analysis. **(H)** Percentage of splenic $CD4^+$Ki-67^+ in different treatment groups analyzed with flow cytometry analysis. **(I)** Percentage of splenic Treg cell in different treatment groups analyzed with flow cytometry analysis. **(J)** Percentage of splenic granzyme B^+ Treg cells in different treatment groups analyzed with flow cytometry analysis. Data expressed as mean ± SEM. Two tailed unpaired t test between groups unless otherwise labeled, * $p<0.05$, ** $p<0.01$. n= 8-13 for each group.

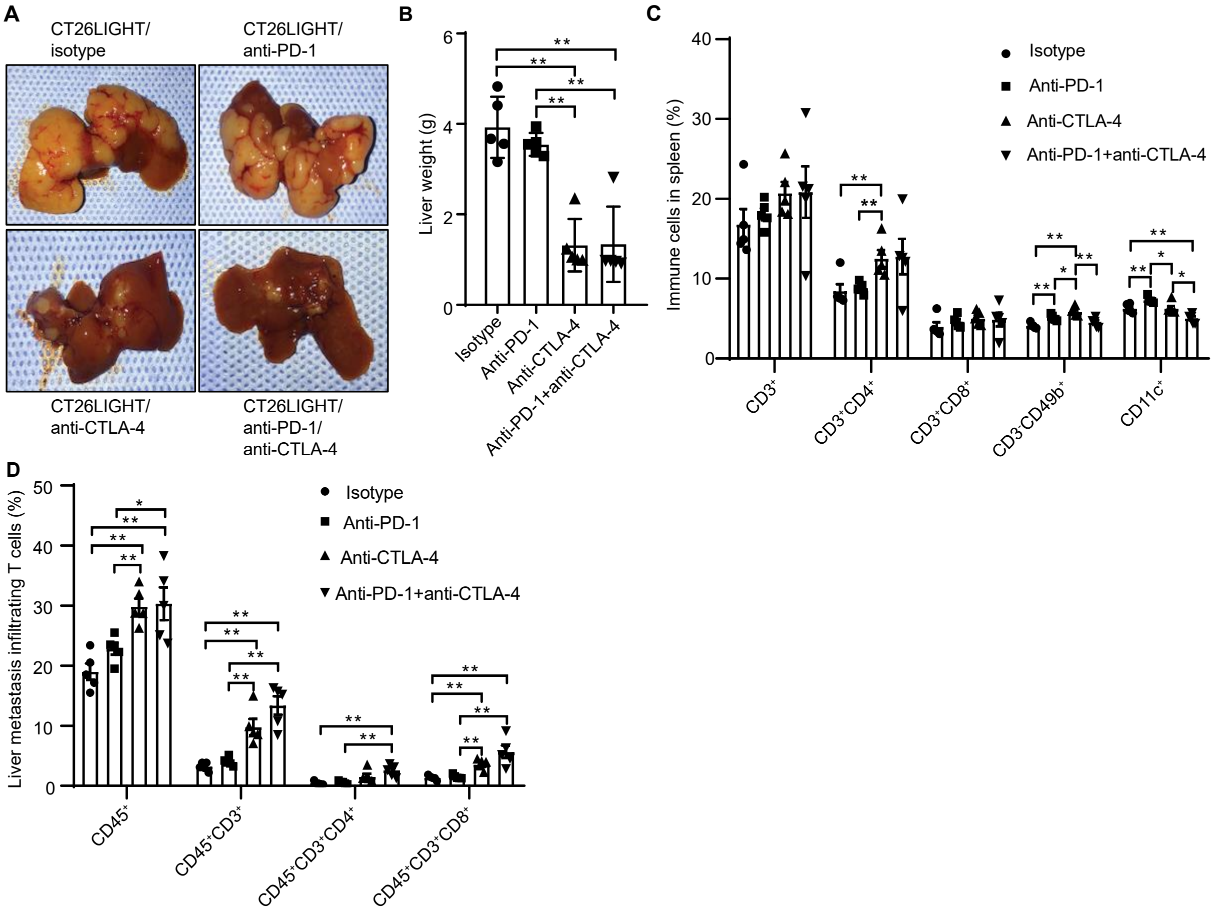

Figure S2. Impact of LIGHT overexpression with combination immune checkpoint blockade therapy (anti-CTLA-4 and anti-PD1) on colorectal liver metastasis development. **(A)** Colorectal liver metastasis (CRLM) were established with LIGHT expressing colorectal cancer cells (CT26LIGHT), and mice were treated with anti-CTLA-4, anti-PD-1, isotype, or both, for four doses starting on day 4 at three day intervals. Representative photos of livers taken on day 14. **(B)** Liver tumor burden from different treatment groups. We have previously shown liver weight to quantitatively correlate with metastatic liver tumor burden based on tumor area measurements in this model (15). **(C)** Flow cytometry results of splenic immune cell populations in mice from different treatment groups. **(D)** Flow cytometry of tumor infiltrating immunocytes in CRLM from different treatment groups. n= 5 per group.

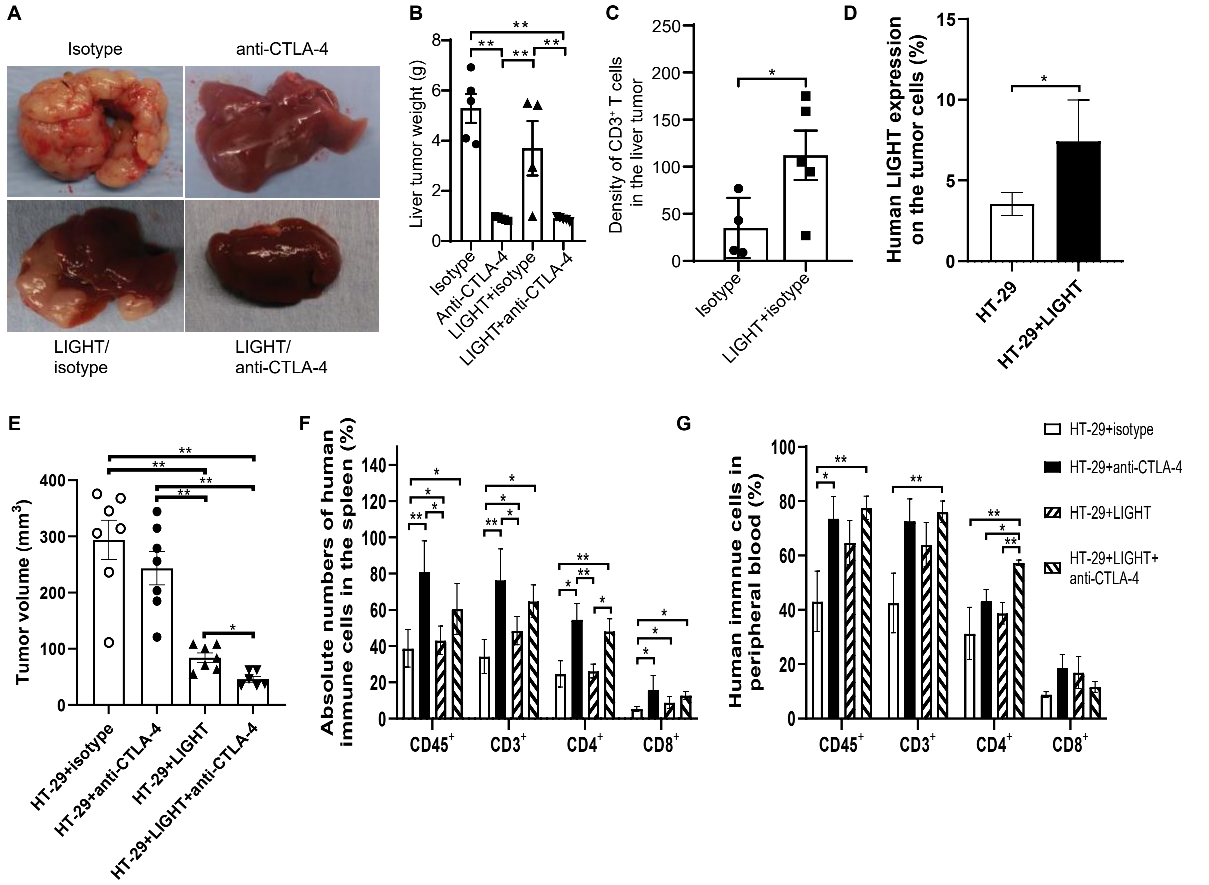

Figure S3. LIGHT overexpression enhances T cell infiltration and anti-CTLA-4 combination therapy disrupts colorectal liver metastases development in MC38 and HT-29 models. **(A)** Isolated CRLM in C57BL/6 mice (n=5 per group) were established as in Figure 1 utilizing MC38 syngeneic colorectal cancer cells. Mice were treated with four doses of anti-CTLA-4 starting on day 3 at three day intervals. Livers were explanted on day 18. **(B)** Liver tumor burden of mice from different treatment groups after sacrifice. We have previously shown liver weight to quantitatively correlate with metastatic liver tumor burden based on tumor area measurements in this model (15). **(C)** Density of $CD3^+$ T cells in metastatic liver tumor tissue from immunochemical staining with mouse anti-CD3. There was no tumor in combination or anti-CTLA-4-treated animals, which was expected due to the known sensitivity of this microsatellite instable murine cell line to anti-CTLA-4 monotherapy. **(D)** Human LIGHT expression as analyzed by flow cytometry in tumor cell lines used in the experiments shown in E-G. n=7 per group. **(E)** Wild-type or LIGHT inducible HT-29 human colorectal cells were inoculated into the flank of NSG mice reconstituted with matched human PBMC. Mice received systemic anti-CTLA-4 or isotype control. LIGHT expression was induced on day 10 when the tumor volume reached 150 to 200 mm^3. Mice were administered PBS, isotype control, and anti-CTLA-4 at three-day intervals and mice were sacrificed on day 26. **(F)** Flow cytometry results of splenic immune cell populations in mice from different treatment groups. **(G)** Flow cytometry

results of peripheral blood immune cell populations in mice from different treatment groups. Data is shown as mean ±SEM, using a two tailed unpaired t test between groups, * $p<0.05$, ** $p<0.01$.

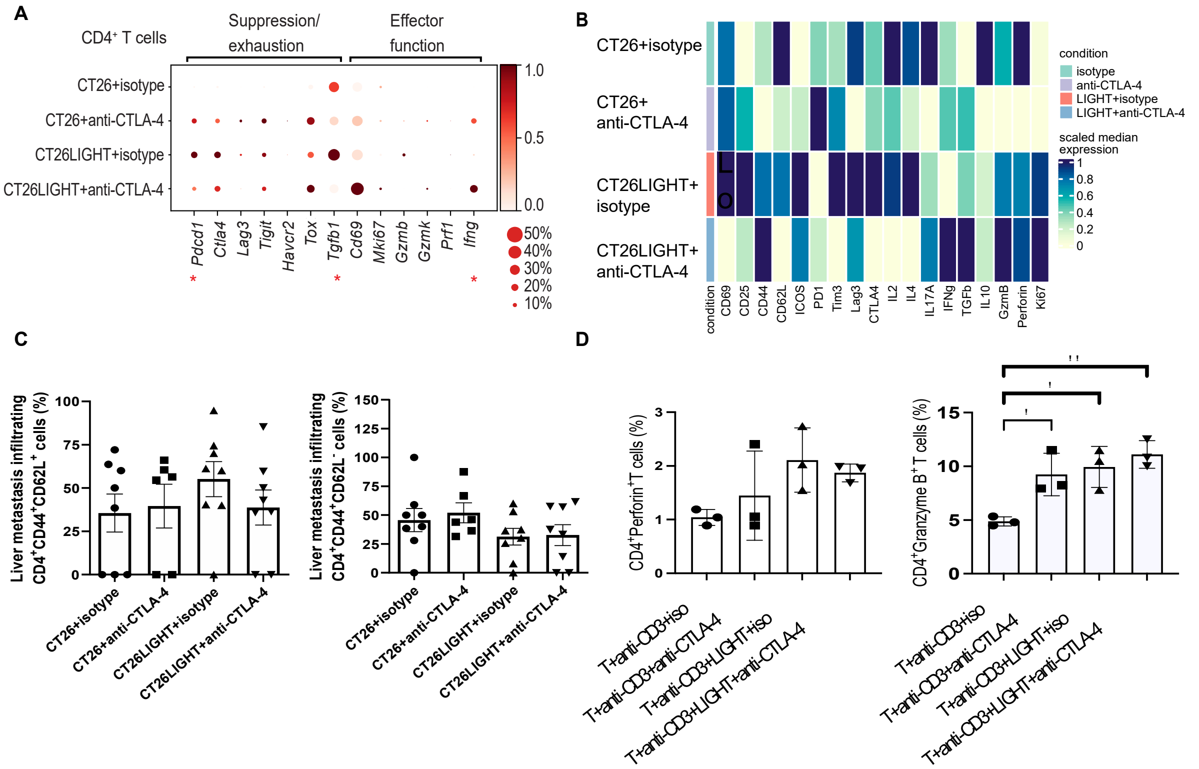

Figure S4. Combination treatment promotes tumor infiltrating CD4^{+} T cells activation and cytokine production. (A) Dot plot shows the expression levels of genes related to tumor infiltrating CD4^{+} T cell activation, exhaustion, cytokine production and function analyzed with scRNAseq. In the expression plot, * indicates any statistical comparisons between the CT26LIGHT + anti-CTLA-4 group and any of the other groups. n=3 per group. **(B)** The heat map shows expression levels of a set of proteins associated with CD4^{+} T cell activation, proliferation, cytokine production and function in the CRLM analyzed with CyTOF analysis. n=3 per group. (**C**) The percentage of CD44^{+}CD62L^{+} cell (T central memory) and CD44^{+}CD62L^{-} cell (T effector memory) out of CD4^{+} T cells in the CRLM from flow cytometry analysis (n=6 to 8 per group), mean ±SEM is shown on plots. (**D**) The percentage of Perforin^{+} and Granzyme B^{+} CD4^{+} T cells after *in vitro* stimulation with LIGHT and anti-CTLA-4.

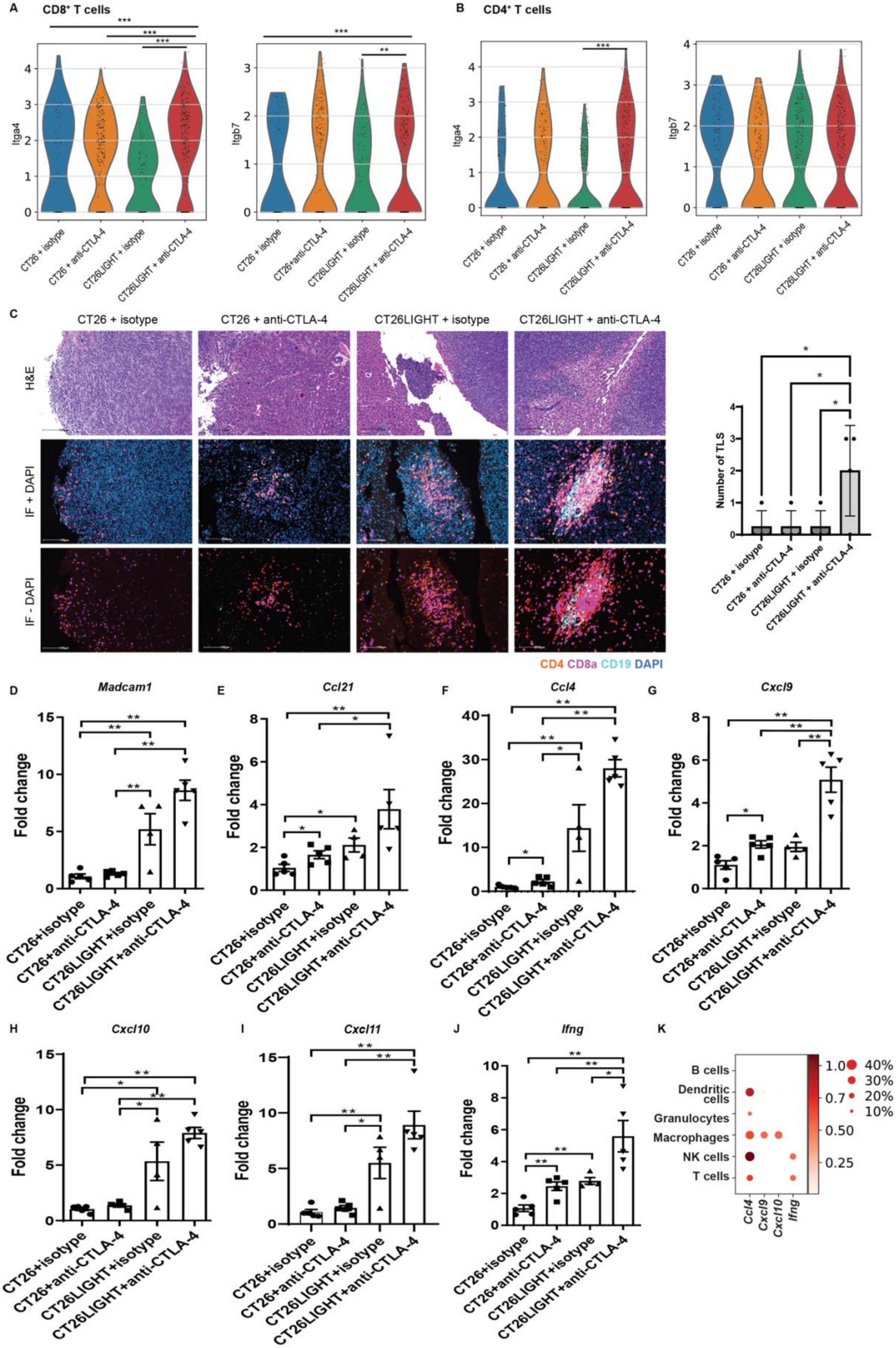

A
CD8⁺ T cells
B
CD4⁺ T cells
Itga4
Itgb7
CT26 + isotype
CT26 + anti-CTLA-4
CT26LIGHT + isotype
CT26LIGHT + anti-CTLA-4
C
H&E
IF + DAPI
IF - DAPI
Number of TLS
CD4 CD8a CD19 DAPI
D
Madcam1
E
Ccl21
F
Ccl4
G
Cxcl9
H
Cxcl10
I
Cxcl11
J
Ifng
Fold change
CT26+isotype
CT26+anti-CTLA-4
CT26LIGHT+isotype
CT26LIGHT+anti-CTLA-4
K
B cells
Dendritic cells
Granulocytes
Macrophages
NK cells
T cells
Ccl4
Cxcl9
Cxcl10
Ifng
40%
30%
20%
10%

Figure S5. Combination treatment enhances expression of immune cell adhesion molecules and chemokines involved in T cell trafficking. (A-B) The violin graphs show the expression of *Itga4* and *Itgb7* in (A) $CD8^+$ and (B) $CD4^+$ T cells in the CRLM analyzed with scRNAseq analysis. n=3 per group. All p values adjusted for false discovery rate. * = p<0.05, **= p<0.01 and ***=p<0.001. **(C)** Immunofluorescent (IF) staining for CD8a, CD4, and CD19 demonstrates the formation of tertiary lymphoid structures (TLS), representative fields are shown as H&E stained sections, with and without DAPI overlaid with other IF staining. Staining was performed on n=4 tumors per experimental group and TLS number per sample was quantified. *p<0.05. **(D-I)** Expression of (D) *Madcam1*, (E) *Ccl21*, (F) *Ccl4*, (G) *Cxcl9*, (H) *Cxcl10*, (I) *Cxcl11* and (J) *Ifng* was assessed using qPCR of CT26 or CT26LIGHT CRLM tumors treated with either isotype control or anti-CTLA-4. *<p0.05. **p<0.01. n=5 per group. **(K)** Expression of *Ccl4, Cxcl9, Cxcl10,* and *Ifng* by immune cell type in the CRLM analyzed with scRNAseq analysis. Color of the dot corresponds with mean expression within each category and the size of the dot corresponds to the fraction of cells expressing the transcript. Transcript for *Madcam1*, *Ccl21*, and *Cxcl11* were not detectable by scRNAseq.

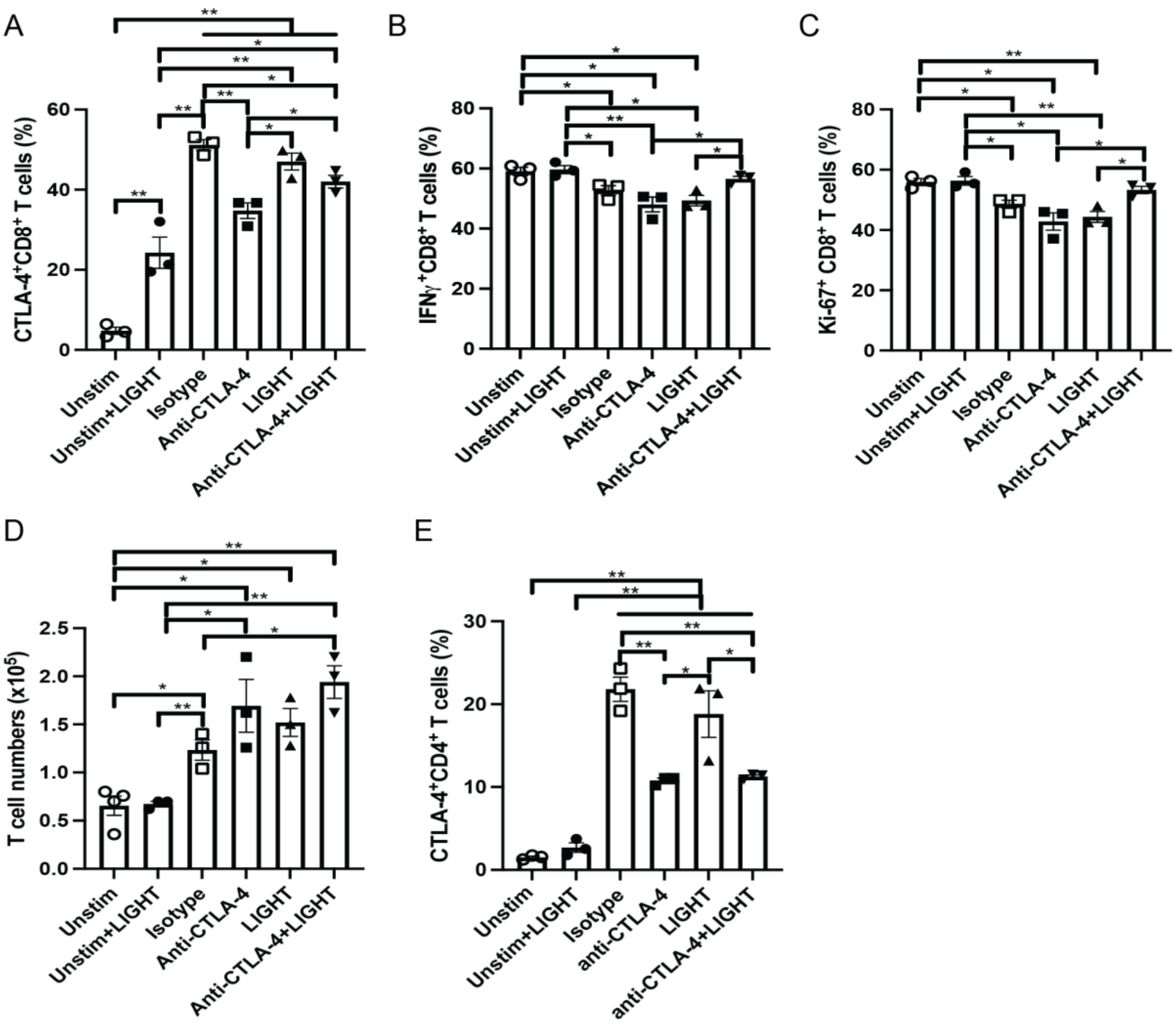

Figure S6. LIGHT and anti-CTLA-4 combination therapy *in vitro* decreases markers of T cell exhaustion. **(A-C)** The percentage of (A) CTLA-4$^+$, (B) IFNγ$^+$, and (C) Ki67$^+$ of CD8$^+$ T cells, **(D)** total T cell numbers, and **(E)** the percentage of CTLA-4$^+$ CD4$^+$ T cells, via flow cytometry analysis (n=3 wells per group) following co-culture for 8 days in the indicated culture conditions. *$p<0.05$, **$p<0.01$. Unstim = unstimulated control.

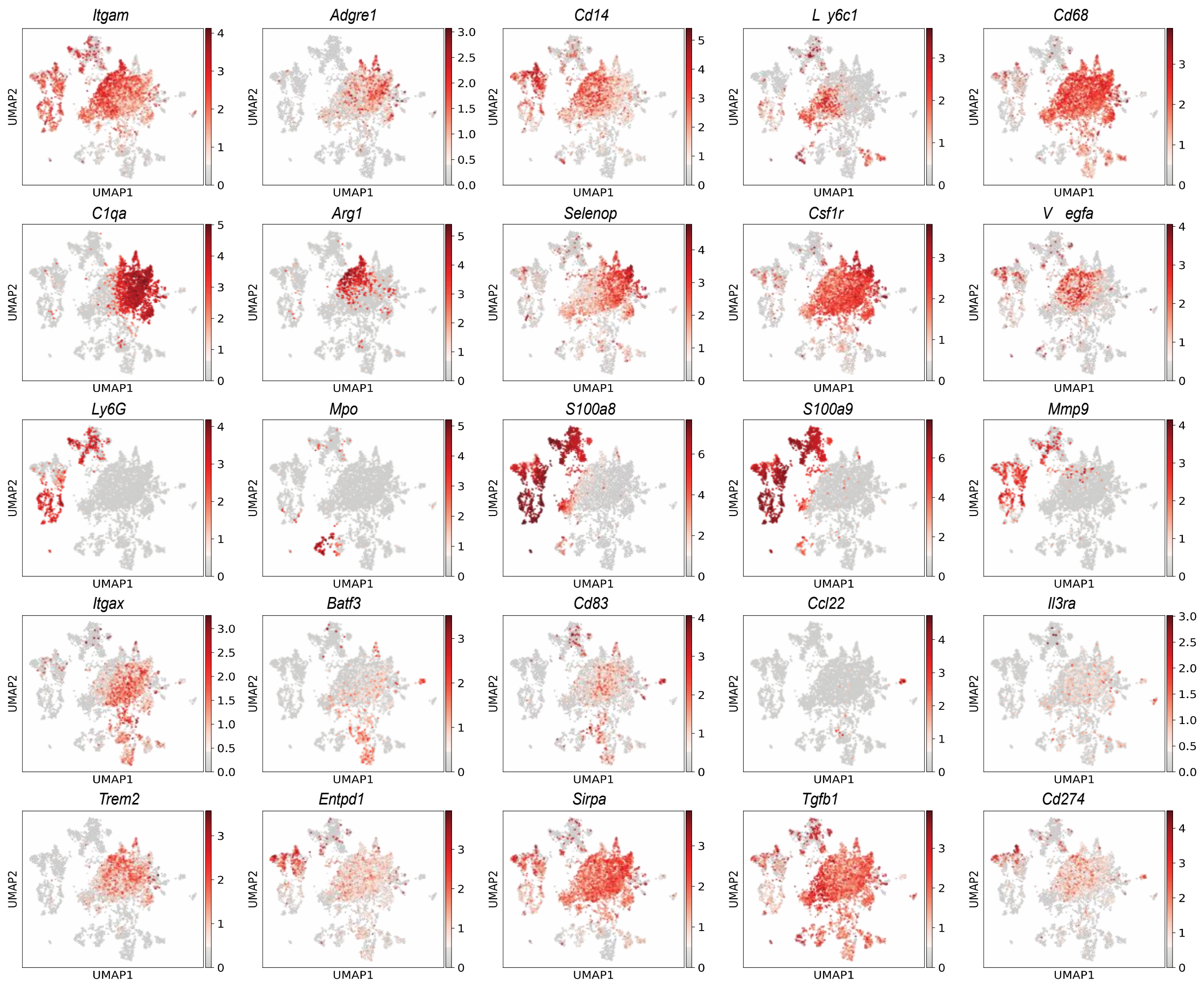

Figure S7. Gene expression characterizing myeloid cells in the tumor microenvironment. RNA expression used to classify macrophage, MDSC, neutrophil and DC cells is shown overlaid on myeloid cell UMAP plot.

Supplementary Table S1. Antibody list for mass cytometry.

Target	Clone	Metal Tag	Stain	Dilution	Source	Cat#
CD45	30-F11	89Y	Surface	100	Fluidigm	3089005C
CD3	145-2C11	152Sm	Surface	100	Fluidigm	3152004C
CD4	RM4-5	145Nd	Surface	100	Fluidigm	3145002C
CD8	53-6.7	146Nd	Surface	100	Fluidigm	3146003C
CD19	6D5	149Sm	Surface	100	Fluidigm	3149002C
CD25	3C7	151Eu	Surface	100	Fluidigm	3151007C
CD69	H1.2F3	143Nd	Surface	100	Fluidigm	3143004C
CD44	IM7	150Nd	Surface	100	Fluidigm	3150018C
CD62L	MEL-14	160Gd	Surface	100	Fluidigm	3160008C
ICOS	C398.4A	175Lu	Surface	100	Fluidigm	3175039C
Foxp3	FJK-16s	165Ho	Intracellular	100	Fluidigm	3165024C
Ki-67	B56	168Er	Intracellular	100	Fluidigm	3168007C
IL-2	JES6-5H4	144Nd	Intracellular	100	Fluidigm	3144002C
IL-4	11B11	166Er	Intracellular	100	Fluidigm	3166003C
IL-17A	TC11-18H10.1	169Tm	Intracellular	100	Fluidigm	3169005C
IFNγ	XMG1.2	176Yb	Intracellular	100	Biolegend	201176A
IL-10	JES5-16E3	158Gd	Intracellular	100	Fluidigm	3158002C
TGFβ	TW7-16B4	164Dy	Intracellular	100	Fluidigm	3164014C
TNFα	MP6-XT22	141Pr	Intracellular	100	Fluidigm	3141013C
Granzyme B	GB11	171Yb	Intracellular	100	Fluidigm	3171002C
Perforin	OMAK-D	172Yd	Intracellular	100	Fluidigm	3172018C
PD-L1	10F.9G2	153Eu	Surface	100	Fluidigm	3153016C
PD-1	29F.1A12	159Tb	Surface	100	Fluidigm	3159024C
CTLA-4	UC10-4B9	154Sm	Surface	100	Fluidigm	3154008C
LAG-3	C9B73	174Yb	Surface	100	Fluidigm	3174019C
TIM-3	RMT3-23	162Dy	Surface	100	Fluidigm	3162029C
CD40	HM40-3	161Dy	Surface	100	Fluidigm	3161020C
I-A/I-E	M5/114.15.2	209Bi	Surface	100	Fluidigm	3209006C
Live/Dead	N/A	194Pt	Surface	2000	Fluidigm	201064

Supplementary Table S2. Antibody list for flow cytometry.

Target	Clone	Source	Cat#
CD45-FITC	30-F11	TONBO Bioscience	35-0451-U100
CD3-APC	17A2	TONBO Bioscience	20-0032-U100
CD4-violetFluor™ 450	GK1.5	TONBO Bioscience	75-0041-U100
CD8-PE-Cy7	53-6.7	TONBO Bioscience	60-0081-U100
CD4-PE	GK1.5	TONBO Bioscience	50-0041-U100
CD25-violetFluor™ 450	PC61.5	TONBO Bioscience	75-0251-U100
CD44-PE	IM7	TONBO Bioscience	50-0441U100
CD62L-PerCP-Cy5.5	MEL-14	TONBO Bioscience	65-0621-U100
CD86-APC	GL-1	TONBO Bioscience	20-0862-U100
MHCII-APC-Cy7	M5/114.15.2	TONBO Bioscience	25-5321-U100
B220-violetFluor™ 450	RA3-6B2	TONBO Bioscience	75-0452-U100
CD3-APC-Cy7	17A2	TONBO Bioscience	25-0032-U100
CD11b-PE-Cy7	M1/70	TONBO Bioscience	60-0112-U100
Ly6G-PE	RB6-8C5	TONBO Bioscience	50-5931-U100
F4/80-APC-Cy7	BM8.1	TONBO Bioscience	25-4801-U100
PD-L1-PE-Cy5	10F.9G2	TONBO Bioscience	65-1243-U100
PD-1-APC	RMP1-30	TONBO Bioscience	20-9981-U100
CTLA-4-PE	UC10-4F10-11	TONBO Bioscience	50-1522-U100
LAG-3-PE	C9B7W	BioLegend	125208
TIM-3-APC	B8.2C12	BioLegend	134008
Perforin-PE	S160098	BioLegend	154406
Granzyme B-PerCP-Cy5.5	QA16A02	BioLegend	372212
Ki-67-PE	11F6	BioLegend	151210
ICOS-PE	15F9	BioLegend	107706
CD49-PE-Cy7	DX5	BioLegend	108922
Human IgG-PE		Invitrogen	12-4998-82
Foxp3-APC	FJK-16s	Invitrogen	17-5773-82
CD11c-eFluor™ 450	N418	Invitrogen	48-0114-82
CD69-PerCP-Cy5.5	H1.2F3	eBioscience	45-0691-82
CD80-PE	16-10A1	BD Pharmingen	553769

Supplementary Table S3. RT-PCR primer sequences.

Gene	Forward primer sequence	Reverse primer sequence
Madcam1	5’-GACACCAGCTTGGGCAGTGT-3’	5’-CAG CAT GCC CCG TAC AGA G-3’
Ccl21	5’-AGA CTC AGG AGC CCA AAG CA-3’	5’-GTT GAA GCA GGG CAA GGG T-3’
Ccl4	5’-CAA ACC TAA CCC CGA GCA ACA C-3’	5’-GGT CTC ATA GTA ATC CAT CAC AAA GC-3’
Cxcl9	5’-GCC ATG AAG TCC GCT GTT CT-3’	5’- AGG AGC ATC GTG CAT TCC TT-3’
Cxcl10	5’- GCC GTC ATT TTC TGC CTC AT-3’	5’- GCT TCC CTA TGG CCC TCA TT-3’
Cxcl11	5’-CAG GAA GGT CAC AGC CAT AGC -3’	5’- ACA GCG CCC CTG TTT GAA C-3’
Ifng	5’- TCA AGT GGC ATA GAT GTG GAA GAA-3’	5’- TGG CTC TGC AGG ATT TTC ATG-3’
Gapdh	5’-AAC TTT GGC ATT GTG GAA GG-3’	5’-CAC ATT GGG GGT AGG AAC AC-3’
